# Deep learning-based temporal change detection of broadleaved weed infestation in rice fields using UAV multispectral imagery

**DOI:** 10.3389/fpls.2025.1655391

**Published:** 2025-10-28

**Authors:** Rhushalshafira Rosle, Nik Norasma Che’Ya, Fariq Rahmat, Nur Syazyla Sulaiman, Nurul-Idayu Zakaria, Zulkerami Berahim, Mohd Husni Omar, Mohd Razi Ismail

**Affiliations:** ^1^ Department of Agriculture Technology, Faculty of Agriculture, Universiti Putra Malaysia (UPM), Serdang, Malaysia; ^2^ Department of Electrical and Electronic Engineering, Faculty of Engineering Universiti Putra Malaysia (UPM), Serdang, Malaysia; ^3^ Institute of Tropical Agriculture and Food Security (ITAFoS), Universiti Putra Malaysia (UPM), Serdang, Malaysia; ^4^ Department of Crop Science, Faculty of Agriculture, Universiti Putra Malaysia (UPM), Serdang, Malaysia

**Keywords:** deep learning, change detection, phenotyping technologies, weed infestation, UAV multispectral imagery, precision agriculture

## Abstract

Timely and accurate monitoring of weed infestation is essential for optimizing herbicide application in rice cultivation, particularly within site-specific weed management (SSWM) strategies. Conventional blanket spraying remains widely adopted by farmers, resulting in excessive herbicide usage and increased costs. This study presents a deep learning-based change detection approach to evaluate the temporal dynamics of broadleaved weed infestation in paddy fields. Multispectral imagery was collected using unmanned aerial vehicles (UAVs) over PadiU Putra rice fields, and a Deep Feedforward Neural Network (DFNN) was developed to classify three land cover types: paddy, soil, and broadleaved weeds during the vegetative stage. Post-classification comparison was applied to assess weed infestation rates across multiple Days After Sowing (DAS). The analysis revealed a consistent increase in weed coverage within untreated plots, with infestation rates rising from 40.95% at 34 DAS to 47.43% at 48 DAS, while treated plots remained largely controlled. The change detection maps further enabled estimation of potential herbicide savings through targeted application, indicating a possible reduction of up to 40.95% at 34 DAS. However, continued weed growth reduced this to 37.06%, with an R² of 0.9487, indicating a strong negative correlation between weed coverage and herbicide-saving potential. These findings demonstrate the potential of integrating UAV-based multispectral imaging with deep learning for temporal weed monitoring and precision agriculture applications.

## 1 Introduction

Rice is a major staple crop that feeds billions of people worldwide (Dorairaj and Govender, 2023). However, its productivity is frequently threatened by weed infestation, which competes with rice for essential resources such as nutrients, water, light, and space (Yu et al., 2022). Among various weed types, broadleaved weeds are particularly damaging, especially during both the main and off-seasons. Their rapid growth and adaptability can significantly reduce yields if not effectively controlled (Hazrati et al., 2023).

Previous studies report that uncontrolled weeds can reduce rice yields by up to 80%, depending on infestation severity and management practices (Dudchenko et al., 2021; Rosle et al., 2021). For example, yield losses in Bangladesh range from 20–50% during the winter season and 15–68% during the monsoon (Islam et al., 2021). In California, weed pressure has led to yield losses of up to 69% along with reductions in grain quality and biomass (Karn et al., 2020). The conventional method of blanket herbicide application remains widely practiced but presents several critical limitations. This approach applies to herbicide uniformly, regardless of weed distribution, resulting in excessive chemical usage, environmental pollution, the development of herbicide-resistant weed species, and health risks for farmers (Liu et al., 2021; Ofosu et al., 2023; Ghazi et al., 2023).

In recent years, unmanned aerial vehicles (UAVs) have gained increasing attention in precision agriculture due to their ability to capture high-resolution imagery rapidly and cost-effectively. UAV-based data acquisition facilitates weed mapping, enabling the detection and spatial localization of weed patches. When combined with deep learning (DL) techniques, UAV imagery can be automatically analyzed for efficient and accurate weed detection (Elakya et al., 2022; Aparna et al., 2023; Guo et al., 2024). DL models such as Segmentation Network (SegNet), Pyramid Scene Parsing Network (PSPNet), UNet, and Fully Convolutional Network (FCN) have demonstrated high performance in weed classification tasks, achieving classification accuracy exceeding 90% in paddy field studies (Kamath et al., 2022; Huang et al., 2020). However, most of these studies focus on weed mapping at a single time point. Temporal analysis of weed infestation remains underexplored, particularly when utilizing deep learning in combination with multispectral UAV data (Wang et al., 2022).

Therefore, the integration of remote sensing (RS) techniques with UAVs has enabled high-resolution monitoring of crop and weed dynamics over time. This study adopted multispectral imagery, despite the finer spectral resolution and discrimination capabilities offered by hyperspectral systems. Hyperspectral imagery remains less commonly used due to its higher cost and data complexity (Sulaiman et al., 2022). In contrast, multispectral systems offer a cost-effective alternative with reduced data volume and faster processing times (Celikkan et al., 2025). These sensors capture reflectance across multiple spectral bands, allowing for effective differentiation between crops and weeds based on their unique spectral signatures (Zhang et al., 2025).

According to Seiche et al. (2024), multispectral systems including low-cost configurations can achieve F1-scores ranging from 76% to 82% for weed classification, while high-end systems can reach up to 90% precision. Although comparative studies between multispectral and hyperspectral imaging in weed monitoring remain limited in recent literature (2022–2024), emerging works have begun to address this gap. Busari and Abdulrahman (2025) demonstrated that integrating hyperspectral data with Vision Transformers significantly enhances weed classification accuracy under field variability. Similarly, Che’Ya et al. (2021) showed that while hyperspectral reflectance offers superior spectral discrimination, optimized multispectral bands can achieve comparable detection accuracy with higher spatial resolution. Thus, integrating RS with deep learning (DL) models, such as Deep Feedforward Neural Networks (DFNNs), presents a promising pathway for automating weed detection and classification at high spatial and spectral resolutions (Touvron et al., 2022; Xia et al., 2022).

Beyond spatial weed mapping, understanding temporal changes in weed distribution is crucial for timely intervention and more efficient management. Change detection (CD) techniques identify land cover changes by comparing imagery acquired at different time points (Khelifi and Mignotte, 2020). In agricultural applications, CD can track weed spread, support herbicide decision-making, and enhance site-specific interventions. While several CD studies have applied classical and hybrid DL-based approaches for land use and vegetation monitoring (Sudha and Vaideghy, 2022; Saha et al., 2020b), their application for temporal weed monitoring remains limited. This is partly due to challenges such as:

- Limited availability of high-frequency UAV temporal data.
- High computational demands are associated with processing multispectral imagery.
- The interdisciplinary expertise required across agronomy, machine learning, and remote sensing.

Therefore, this study addresses these gaps by developing a differencing-based change detection framework that utilizes multispectral UAV imagery in combination with a Deep Feedforward Neural Network (DFNN). The framework classifies weed presence across multiple growth stages, analyzes temporal changes in infestation, and estimates potential herbicide reduction through targeted application. The findings contribute to the advancement of site-specific weed management, supporting more sustainable rice production through precision agriculture. In addition, this study makes three key contributions: (i) it introduces a deep learning-based change detection framework that integrates UAV-acquired multispectral imagery with a Deep Feedforward Neural Network (DFNN) to monitor broadleaved weed infestation in rice fields; (ii) it extends beyond static weed mapping by analyzing temporal infestation dynamics across multiple growth stages, providing actionable insights for site-specific herbicide application; and (iii) it demonstrates the practical potential of combining UAV-based remote sensing and deep learning for sustainable precision agriculture, highlighting possible reductions in herbicide use through targeted interventions.

## 2 Materials and methods

### 2.1 Experimental settings

#### 2.1.1 Study area

**Figure 1:**
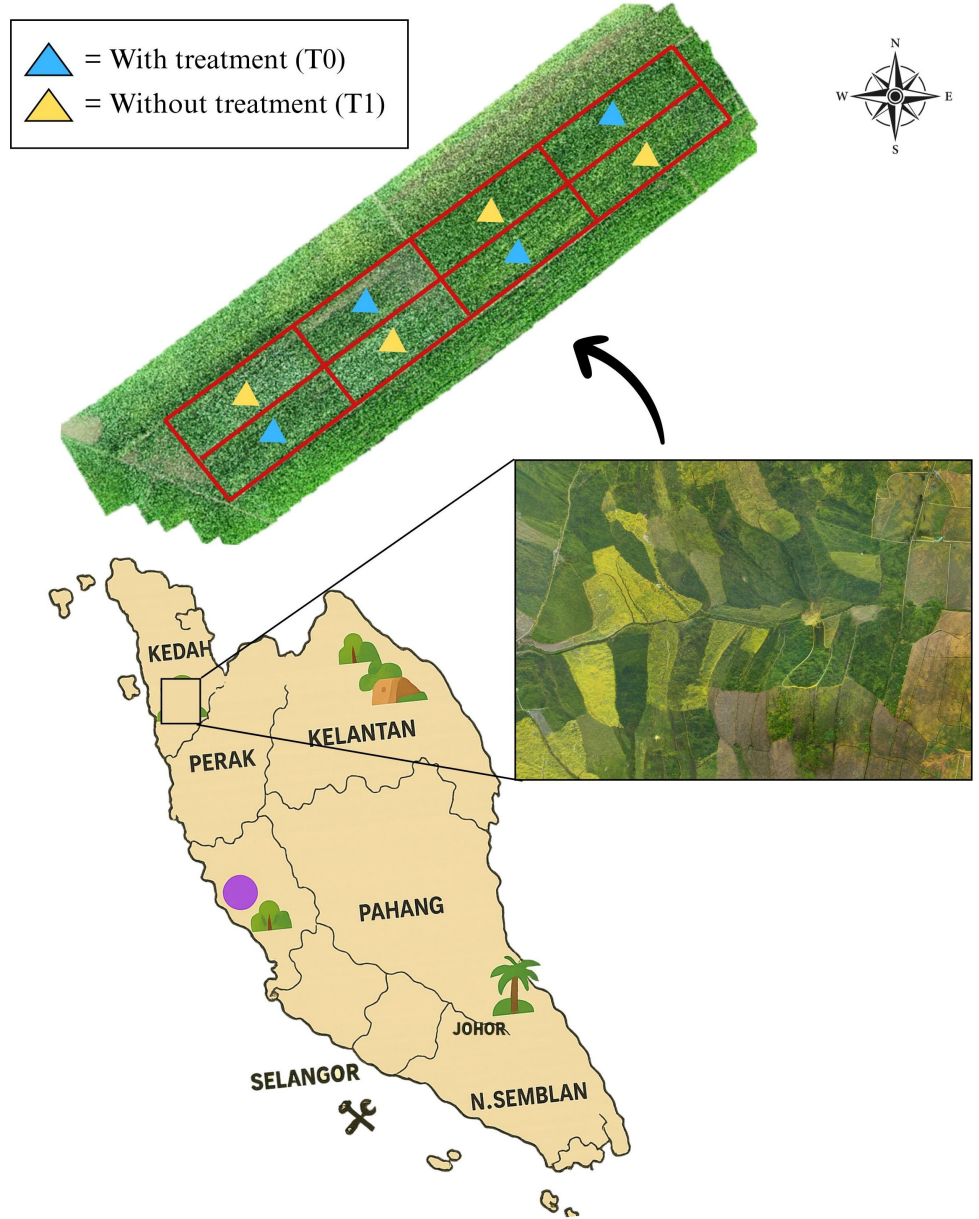
Location of the study at MADA, Tunjang, Jitra, Kedah, Malaysia. The aerial background image of paddy fields (inset) was sourced from Pexels.com (free stock photo, used under Pexels license).

The study was conducted in Tunjang, Jitra, Kedah, Malaysia (6^0^ 16’ 05.8” N, 100^0^ 21’ 10.3” E), covering an area of 0.504 hectares (ha). The location of the study site is illustrated and presented in **Figure 1**. The site was selected in collaboration with the Lembaga Muda Agricultural Development Authority (MADA). The rice variety cultivated in this study was Padi-U Putra, which has a maturation period of 120 days after sowing (DAS) (Berahim et al., 2021). The experiment was conducted during the main cropping season under natural field conditions. Broadleaved weeds were allowed to grow naturally, and their infestation was monitored during the vegetative stage of the Padi-U Putra variety.

#### 2.1.2 Treatment and experimental design

**Figure 2:**
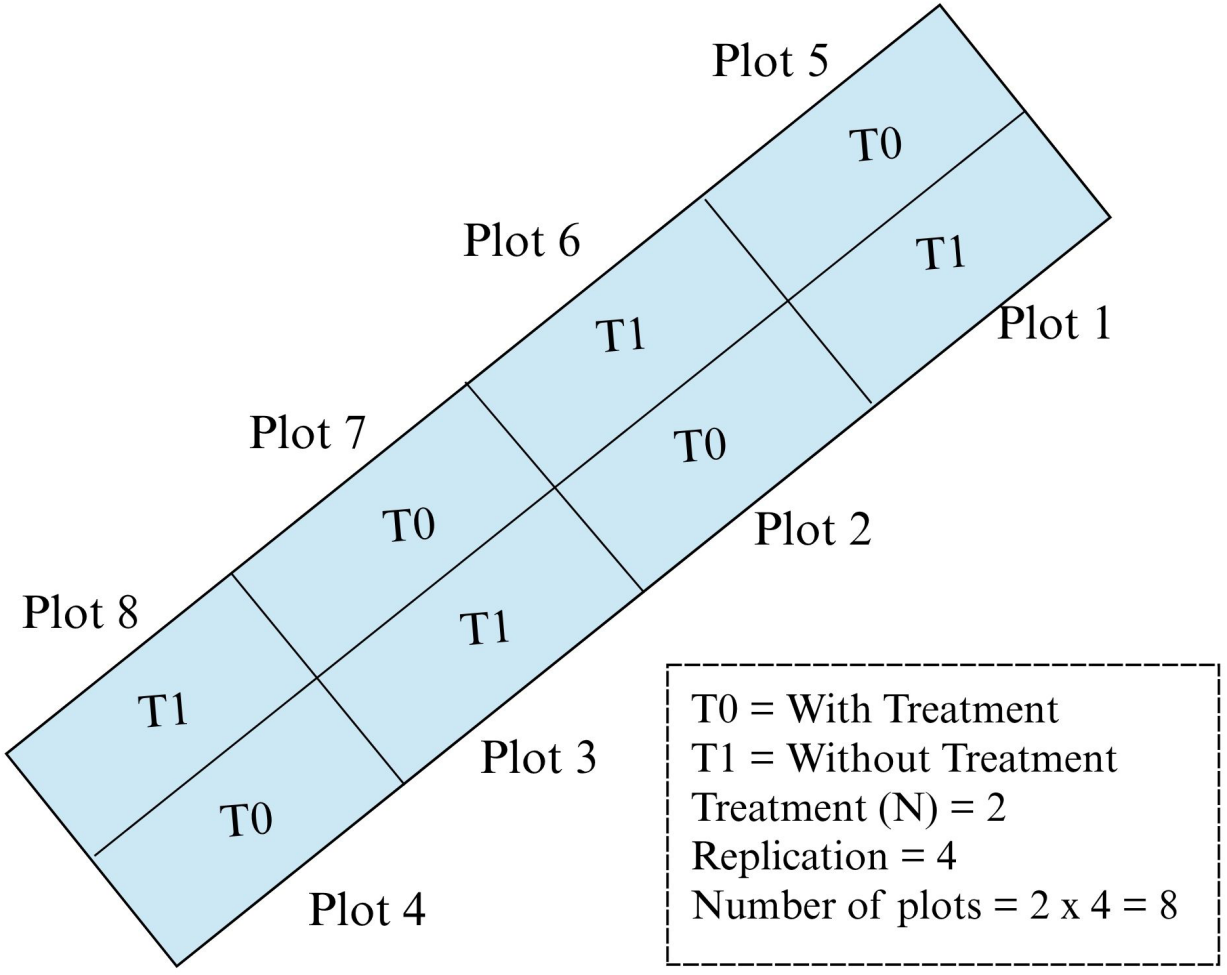
Plot arrangements in the study area.

Two treatments are conducted: with treatment (T0) and without treatment (T1) plots with four replications, which makes the total number of plots in the study plot eight. For treatment plots (T0), fertilizer and herbicide were used. Meanwhile, for without treatment plots (T1), only fertilizer was applied without herbicide application. For this study, farmers used herbicide named 2,4-D amine to control broadleaved weed and fertilizer for paddy as guided in the rice checkbook (DoA, 2022). There are eight plots used in this study, the plot size was approximately 630 m^2^ and the barrier in between the plots is about 30 cm. The plots were arranged in a randomized complete block design (RCBD) as shown in **Figure 2**.

#### 2.1.3 Datasets

Datasets used in this study were captured by a Micasense RedEdge-MX multispectral camera (MicaSense, Seattle, Washington, USA) and attached to a multirotor DJI Inspire 2 UAV (Da-Jiang Innovation Science and Technology Co., Ltd, DJI, China). In this study, DroneDeploy (DroneDeploy, Inc., United States) was used to plan the flying area and pre-set waypoint for autonomous flight mission (Mohd Zaidi and Tahar, 2021).The flying height used in this study was 20m above ground level with a spatial resolution of 0.913 cm. Scene overlap was set to 80% front and 75% side, ensuring sufficient coverage and image alignment, and it was flown with fight speed of 3ms^-1^. All missions were flown between 9.00 am to 11.30 am under clear sky conditions to ensure consistent illumination (Mohidem et al., 2022). This multispectral camera can be captured in five bands: red (R), green (G), blue (B), red-edge (RE), and near infrared (NIR). To ensure radiometric consistency across flight sessions and compensate for varying illumination conditions, radiometric calibration was performed before and after each UAV flight using a calibrated reflectance panel (white reference panel). These reference images were used during processing to correct the raw digital numbers and standardize reflectance values across different dates. The flying season was executed during the Main seasons, and the image acquisition was taken within seven-day intervals. However, due to the COVID-19 outbreak and the implementation of Malaysia’s Movement Control Order (MCO), the data collection process was constrained. As a result, imagery was only acquired at three time points: 30 June 2020 (34 DAS), 7 July 2020 (41 DAS), and 13 July 2020 (47 DAS). While this limits the density of the temporal series, these intervals correspond to critical stages of vegetative growth and weed competition, thereby still providing meaningful insights into infestation dynamics (Safdar et al., 2025).

Pix4D software was used to mosaic the images captured, and their digital numbers were subsequently converted into reflectance values. Following this, geometric registration was applied to ensure pixel-to-pixel correspondence, a crucial step for multi-imagery analysis integration. Given the large size of the original images, approximately 8127 x 6892px, there is a risk of exhausting GPU memory during processing. To mitigate this issue and expedite processing time, as suggested by Huang et al. (2018), each image captured on different dates was subset into eight plots, approximately 2262 x 2091px for each plot. Also, training data normalization is performed to standardize the input features.

#### 2.1.4 Training data collection and preparation

Training samples are essential for classification processing as they require representative samples for each class (Li et al., 2024). Sample data were selected from UAV imagery based on *in situ* observations, focusing on three main classes: paddy, soil and weed. Given the study’s incorporation of two treatments; with treatment (T0) and without treatment (T1), training datasets were collected for each treatment, resulting in two datasets. To ensure temporal representation, training samples were collected from imagery acquired from 34 DAS and 41 DAS. The samples were gathered using random sampling strategies. The input fed into the DFNN model was converted into structured, non-spatial data extracted from each pixel’s reflectance across the Red, Green, Blue, Red Edge, and Near-Infrared (NIR) bands. This format was chosen for its computational efficiency (Li et al., 2024), and it is well-suited for DFNN, which has a simpler architecture, requires fewer computational resources, and is faster to train compared to Convolutional Neural Networks (CNNs). It also suits situations where spatial information is limited or unnecessary (Aydin and Sefercik, 2025).

In addition, training samples were randomly shuffled to remove sequence bias before model training. This pixel-wise, non-spatial format aligned well with the model, which are optimized for structured, tabular data rather than spatial image features. Therefore, following empirical testing and cross-validation to balance model complexity and generalization performance, **Table 1** summarized the total used to train the model.

**Table 1:** The total number of training samples used to train the model.

Data Collection	Multispectral Image Pixel				
	With Treatment (T0)		Without Treatment (T1)		
	Paddy	Soil	Paddy	Soil	Weed
34 DAS	6480	6471	4751	4756	4755
41 DAS	6474	6485	4769	4766	4760
Total sample for each class	12954	12956	9520	9522	9515
**Total training sample used**	**54467**				

Samples were collected from UAV imagery acquired from 34 and 41 DAS under two treatments: with herbicide (T0) and without herbicide (T1).

*DAS, day after sowing.Bold values highlight total number of training sample used to train the model.

### 2.2 Change detection analysis

**Figure 3:**
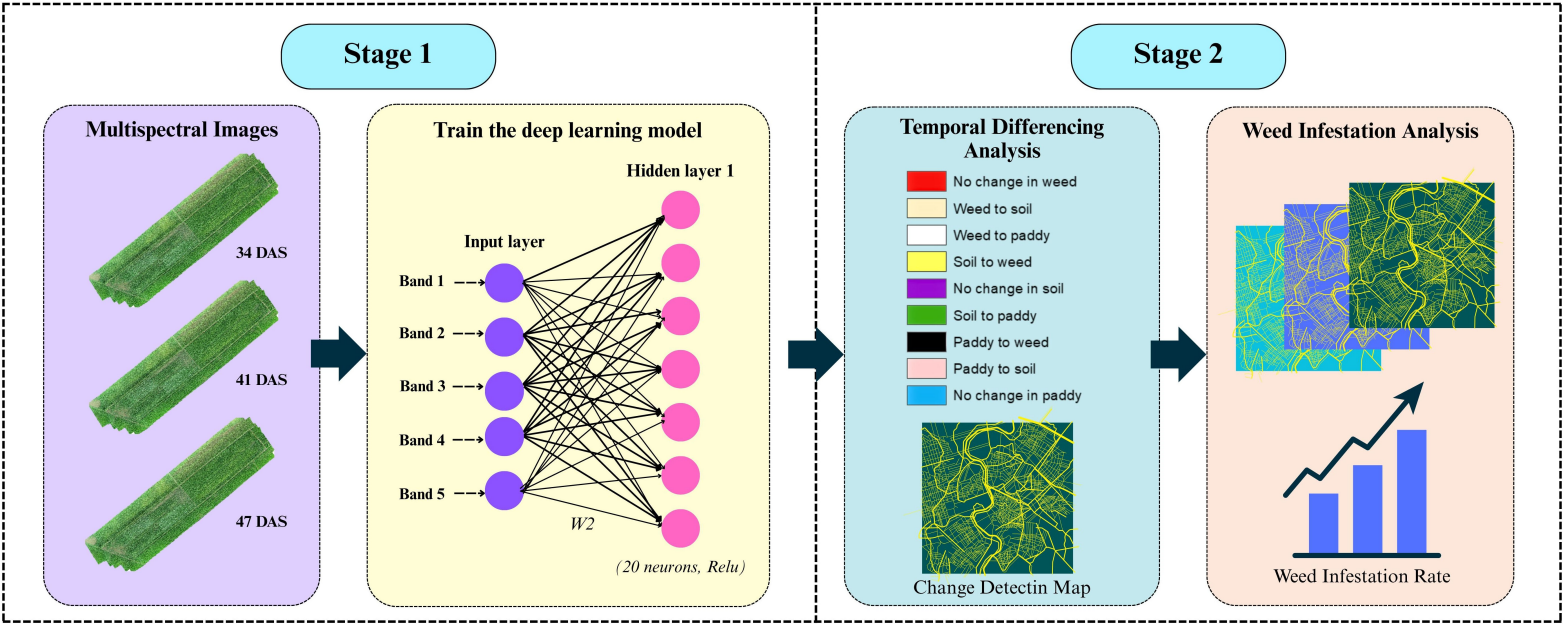
An overview of differencing-based post-classification change detection (CD) framework.

A differencing-based post-classification change detection (CD) framework was employed in this study to evaluate the temporal dynamics of broadleaved weed infestation in rice fields. The approach involved two main stages: multi-temporal image classification using a Deep Feedforward Neural Network (DFNN), followed by temporal differencing of classified images. Therefore, **Figure 3** summarizes the workflow involved in this analysis.

#### 2.2.1 Deep feedforward neural network architecture

**Figure 4:**
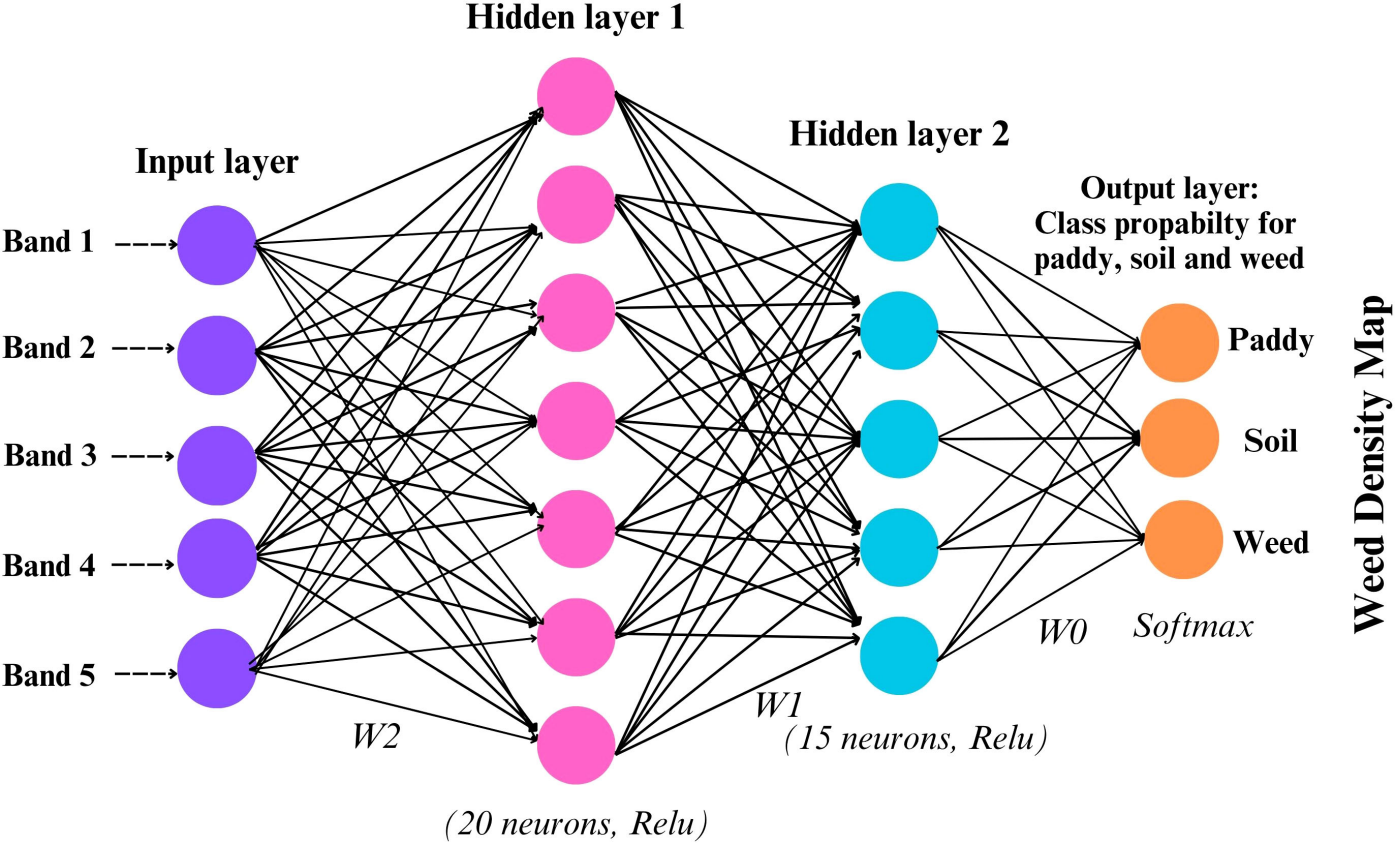
Model architecture of DFNN.

The model was built sequentially using the Keras library in R, with a stack of dense layers forming the core of the architecture. Each dense layer contains a specific number of neurons, determined by the problem’s complexity and dimensionality. For this study, one input layer, two hidden layers, and one output layer were set up in the model. Spectral bands from multispectral imagery, band 1 to band 5 were used as input layers. A DFNN model was trained on annotated samples to classify each pixel into one of three categories: paddy, weed, and soil. Each image was independently classified to produce discrete thematic maps representing the spatial distribution of these classes at each time point. The hidden layer configuration of 20 and 15 neurons was determined through empirical testing and cross-validation to balance model complexity and generalization performance. **Table 2** shown the DFNN architecture and layer configuration that used in this study. All layers are sequentially fully connected (dense) without residual/skip connections: input to Dense(20) to Dropout(0.2) to Dense(15) to Dropout(0.2) to Dense(3, softmax). Larger networks were found to overfit the relatively small training dataset, while smaller configurations underperformed in distinguishing between spectrally similar classes, such as weed, paddy, and soil. **Figure 4** illustrates the model architecture used in this study.

**Table 2:** DFNN architecture and layer configuration.

Layer index	Layer type	Units/shape	Activation	Kernel initializer	Kernel regularizer (L2)	Dropout
0	Input	5 (R,G,B,RE,NIR)	NA	NA	NA	NA
1	Dense	20	ReLU	He normal	L2 (λ = 1e-6)	0.2
2	Dense	15	ReLU	He normal	L2 (λ = 1e-6)	0.2
3	Dense (output)	3	Softmax	Glorot uniform	NA	NA

Due to the labeled dataset, this model can effectively tackle classification problems. Inputs are fully connected to multiple hidden layers, which then predict the outcome. While convolutional neural networks (CNNs) are typically the architecture of choice for image-based classification tasks due to their spatial feature extraction capabilities, this study opted for a DFNN owing to the use of pixel-level spectral inputs from multispectral imagery rather than spatial context. Since the classification task was conducted on a per-pixel basis using reflectance values from the five spectral bands (R, G, B, RE, NIR), the DFNN proved sufficient to capture nonlinear relationships between spectral inputs and land cover classes.

The rectified linear activation function (ReLU) activation function, defined as f(x)=max(0,x) was chosen due to its computational efficiency and ability to mitigate vanishing gradient issues often encountered with sigmoid or tanh functions. ReLU introduces non-linearity while maintaining sparsity in the network, making it well-suited for the high-dimensional input space derived from multispectral imagery. This function is a simple mathematical computation where neurons are activated based on their input; if the output value is less than zero, the neurons are cut off from the network. According to Schmidt-Hieber (2020), one benefit is that it enhances computational efficiency for every parameter modification. The activation function is as shown in Equation 1:


(1) $$f\left(x\right)=\{\begin{array}{c} x\;if\;x\;positive\\ 0\;othewise \end{array}$$

However, when dealing with a fully supervised method, an issue related to overfitted always occurs whenever the supply of training samples is limited (Huang et al., 2018). Therefore, to mitigate this problem, the dropout regularization and L2 regularization technique was applied after each dense layer. Dropout effectively introduces noise during training by randomly setting a portion of input units to zero, preventing the model from depending too much on any feature, which will increase its performance on unseen data (Salehin and Kang, 2023). A dropout rate of 0.2 was applied after each hidden layer, selected based on validation performance. This rate introduces mild stochastic regularization, balancing between reducing co-adaptation of neurons and preserving the learning capacity. Meanwhile, L2 regularization will achieve a balance between generalization and model complexity, incorporating a penalty element into the loss function (Venkatesh et al., 2023). In this study, L2 was applied to the kernel weight of the dense layer, effectively penalizing large weights during training and thus reducing overfitting. The value of λ was tuned to ensure that the network remained expressive without becoming overly sensitive to noise in the training data. Weights were initialized using the He normal initializer for hidden layers to match ReLU activations. The output layer used Glorot uniform initialization. The model optimization was conducted using the Adam optimizer, which combines the benefits of both AdaGrad and RMSProp. Adam adapts learning rates for each parameter and accelerates convergence, particularly in sparse gradients scenarios. The optimizer was configured with a learning rate of 0.005 and default beta parameters $(\beta_{1}=0.9,\;\beta_{2}=0.999$ ). Therefore, the L2 regularization function can be described as shown in Equation 2 below:


(2) $$Cost=Loss+\;\lambda \sum_{i=1}^{n}w_{i}^{2}$$

Where:

Loss = original loss
W_i_ = model weight
λ = strength of regularization

We performed limited empirical tuning on the validation set with early stopping to reduce the search burden. Candidate values tested included neurons per hidden layer {10, 20, 50}, learning rates {1e-6,1e-4, 1e-3, 5e-3, 1e-2}, batch sizes {16, 32, 64}, L2 λ {1e-5, 1e-4, 1e-3}, and dropout rates {0.1, 0.2, 0.4}. Models were trained for up to 200 epochs with early stopping (patience = 10) monitoring validation loss and restoring the best weights. Selection was based on lowest validation categorical cross-entropy (tie-broken by validation F1 for the weed class). The selected hyperparameters: the number of neurons, learning rate, number of epochs, and batch size are summarized in **Table 3**.

**Table 3:** Model hyperparameters and tested values of the DNN model.

Hyperparameters	Tested value
Number of neurons	20 and 15
Learning rate	0.005
Number of epochs	100
Batch size	32

The classification problem addressed in this study is fundamentally a pixel-wise spectral discrimination task: each observation is a five-band vector [R, G, B, RE, NIR] measured at a single date, and the objective is to assign a class label to that vector. Under these conditions, a fully connected feedforward network (DFNN) is an appropriate and efficient choice. DFNNs directly model relationships between the spectral bands for each pixel without introducing additional spatial or temporal structure; they therefore require fewer parameters than patch-based convolutional models and are typically more sample-efficient when training labels are associated with individual pixels rather than image patches. These characteristics reduce the risk of overfitting for datasets with limited labelled samples and simplify deployment across large orthomosaics because inference operates on independent per-pixel vectors.

By contrast, convolutional neural networks (CNNs) are designed to exploit local spatial structure and texture. A CNN requires patch-based inputs (for example, 5\times5 or 11\times11 pixel windows) so that spatial filters can learn patterns in the neighbourhood of each pixel. This is advantageous when spatial context, for example canopy texture, row structure, or object morphology, carries discriminative information that is not present in single-pixel spectra. Recurrent models (RNNs, temporal 1D-CNNs, or temporal transformers) are appropriate when the sequence of observations for a pixel across time is the model input and when temporal dynamics must be modeled directly (for example, phenological trajectories or multi-date time series where each pixel has a labelled temporal profile). Our workflow, however, treats each date independently and relies on post-classification differencing to identify temporal change. Thus, temporal sequence models were not required for the present study.

Given these differences, the DFNN is the simplest model consistent with our data representation and study objectives: it matches the per-pixel input format, keeps the parameter count low relative to patch-based CNNs, and is computationally efficient for whole-orchomosaic inference. We acknowledge that if (a) spatial context were required to disambiguate spectrally similar classes, or (b) labelled temporal sequences per pixel were available and temporal dynamics were to be modeled end-to-end, then patch-based CNNs or temporal models (1D-CNN/RNN/transformer) would be better suited. In those scenarios we recommend either a patch-based CNN or a hybrid architecture that combines convolutional feature extraction with fully connected classification layers, or a temporal model that ingests per-pixel sequences across dates.

#### 2.2.2 Applying DFNN to time-series images

The workflow for applying the trained dense feedforward neural network (DFNN) to time-series orthomosaics follows a strict sequence of preprocessing, inference and post-processing steps designed to preserve temporal consistency and to make the procedure reproducible. Below we describe this procedure in a manner appropriate for the Methods section so that other researchers can re-run the classification and change-detection pipeline using the same inputs, scaler and model weights.

All imagery are first brought to a common radiometric and geometric reference. Radiometric calibration converts recorded digital numbers to surface reflectance using instrument calibration factors and, where available, reflectance-panel measurements captured at each flight. Any vignetting, illumination or simple atmospheric corrections applied during training must be applied here as well so that spectral values are comparable across dates. Geometric registration is then performed to co-register all orthomosaics to a single coordinate reference system and grid (same resolution and CRS), using ground control points or robust image-based tie-point matching; the objective is that a single pixel location corresponds to the same ground point for every date.

Before model inference, quality control masks are applied to all orthomosaics to exclude non-crop areas, image borders, cloud and shadow regions, and any nodata pixels. The policy for masked pixels (e.g., skip prediction or assign nodata) should be documented. For every valid pixel location (i, j) the spectral input vector is assembled in the same band order used for training: R,G,B,RE,NIR, R, G, B, RE, NIR, R,G,B,RE,NIR. To preserve the input distribution expected by the DFNN, each band is standardized by the training-set. It is essential that the same scaler. The same training means, and standard deviations will be used for all dates, ensuring the model receives input on a consistent scale.

Inference is performed by loading the trained DFNN weights and performing predictions in memory-efficient batches over the orthomosaic. For each input vector the network returns class probabilities; the per-pixel label is assigned as the class with maximum probability (argmax). Optionally, the maximum probability can be saved as a per-pixel confidence map to support later uncertainty analyses. Predictions should be exported as raster layers (probability and class rasters) for each date to allow reproducible downstream processing.

Post-processing is applied to reduce speckle and remove spurious small objects: typical operations include connected-component filtering (removing objects below a documented minimum area), median or majority filtering with specified kernel sizes, and conservative morphological smoothing. All parameters used for these operations must be reported. After producing discrete thematic maps for each date, pairwise post-classification differencing is applied (for example, 34 vs 41 DAS and 34 vs 47 DAS) to construct a transition matrix of change categories (no-change, emergence, disappearance, and class-to-class transitions). Class codes must be consistent across dates to ensure the differencing operation is meaningful.

Final area estimates and herbicide requirement computations are obtained by converting pixel counts to ground area (pixel_count × pixel_area) and then applying the domain-specific formulas given in Equations 10–15. The treatment of masked or nodata pixels when summing totals (excluded, interpolated, or proportionally scaled) must be explicitly stated in results and tables. In our procedure the same trained model weights were applied to all dates without per-date retraining; where flight radiometry varies markedly between dates, the reflectance-panel normalization described above should be applied prior to standardization.

#### 2.2.3 Training and validation approach

To ensure the model was trained effectively, performance-optimized, and stable before deployment, the training samples collected will be randomly split into three datasets: training, validation, and test datasets. The model will use the training sample to fit the model. Meanwhile, the validation sample will be used to evaluate the model’s performance and to tune the hyperparameters. Lastly, the test sample dataset will be used to evaluate the model’s overall performance and identify overfitting or underfitting issues. Therefore, for this study, the training samples will be randomly split into 60% (training), 20% (validation), and 20% (testing) (Bonafilia et al., 2020).

The model’s performance will be assessed using eight common metrics: overall accuracy (Equation 3), Kappa Coefficient (Equation 4), categorical cross-entropy loss function (CCE) (Equation 5), Mean Squared Error (MSE) (Equation 6), confusion matrix, precision (Equation 7), recall (Equation 8) and F1-scores (Equation 9). They can be defined as follows:


(3) $$Overall\;accuracy=\frac{\sum_{a=1}^{U}c_{aa}}{Q}\times 100\%$$

where:

*Q* = total number of pixels.
*U* = total number of classes.

(4) $$Kappa\;coefficient,\;K=\frac{\sum_{a=1}^{U}\frac{c_{aa}}{Q}-\sum_{a=1}^{U}\frac{c_{a}.c_{a}}{Q^{2}}}{1-\sum_{a=1}^{U}\frac{c_{a}.c_{a}}{Q^{2}}}\times 100\%$$

where:

*c_a_* = row sums.

(5) $$Cross-entropy\;loss\;\left(CCE\right)=-\frac{1}{N\;}\sum_{i=1}^{N}\sum_{j=1}^{C}y_{ij\;}\text{log}\left(p_{ij}\right)$$

where

*N* = number of samples
*C* = number of classes
*y_ij_* = true label
*p_ij_* = the predicted probability of the true class

(6) $$Mean\;square\;error\;\left(MSE\right)=\frac{1}{n\;}\sum_{i=1}^{N}{\left(y_{i}-{\hat{y}}_{i}\right)}^{2}$$

where

$y_{i}$ = actual (true) value

${\hat{y}}_{i}$ = the predicted value

In addition, to assess classification performance, we computed standard metrics derived from the confusion matrix: precision (Equation 7), recall (Equation 8) and F1-scores (Equation 9). These were calculated for each class using the following formulas:

Let *TP*, *FP*, and *FN* denote true positives, false positives and false negatives, respectively, for a given class *I’*


(7) $$Precision_{i}=\frac{TP_{i}}{TP_{i}+FP_{i}}$$

(8) $$Recall_{i}=\frac{TP_{i}}{TP_{i}+FN_{i}}$$

(9) $$F1_{i}=2\;\times \;\frac{Precision_{i}\;\times \;Recall_{i}}{Precision_{i}+Recall_{i}}$$

These metrics were computed for each class using a one−vs−all approach, providing a more granular evaluation than overall accuracy alone. The resulting per class metrics are summarized in **Table 4**, and the confusion matrices used to derive these values are presented in **Table 5**.

**Table 4:** Overall accuracy and class-specific accuracy.

DAS	Treatment plot	Metric	Training	Validation	Testing
34 and 41	With Treatment (T0)	Accuracy	0.99	0.9893	0.9906
		Loss	0.0287	0.0264	0.0224
		MSE	0.0074	0.0077	0.0066
		Kappa	0.9812		
	Without Treatment (T1)	Accuracy	0.9729	0.98	0.9793
		Loss	0.0791	0.067	0.0597
		MSE	0.0135	0.0104	0.0108
		Kappa	0.9789		

**Table 5:** Confusion matrix for classification under; (a) with-treatment (T0) and (b) without-treatment (T1) conditions.

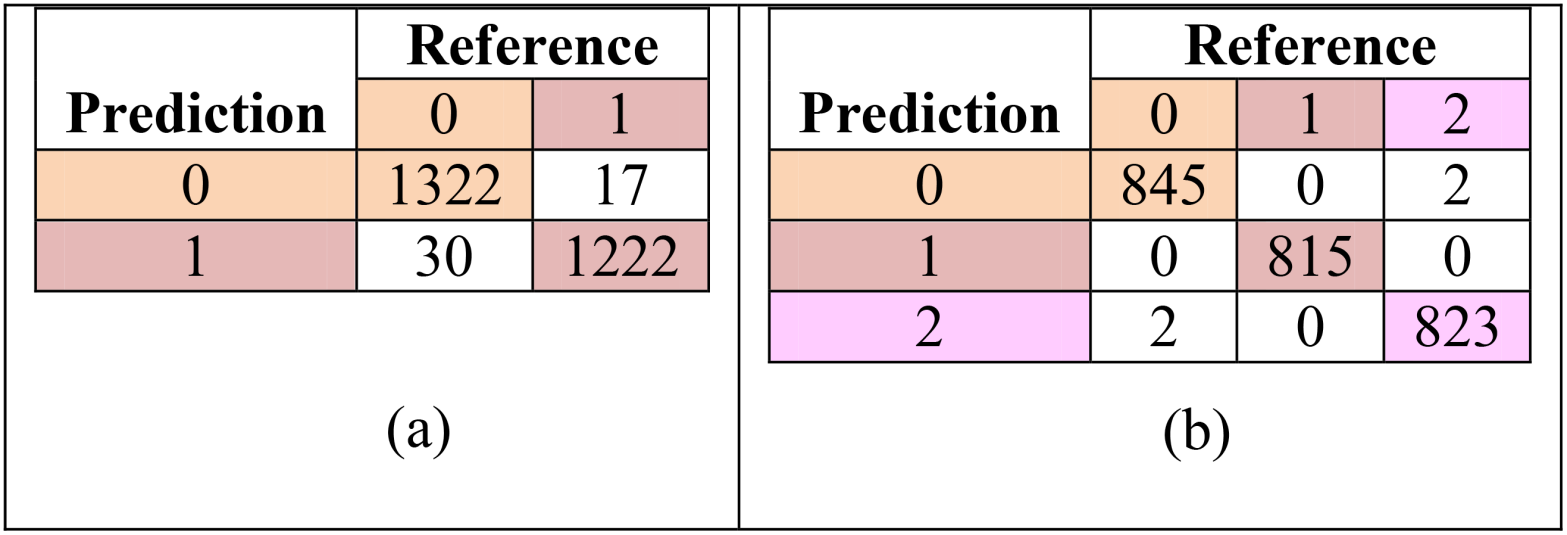

#### 2.2.4 Temporal differencing of classified maps

Following classification, the output maps were analyzed using a class-based differencing technique. This is the best combination since DL-based supervised change detection already produces an accurate result. Meanwhile, pixel differentiation is the most straightforward approach, which highlights the areas of change and calculates the differences between classes that are already classified. In this case, by deep learning models (Asokan and Anitha, 2019; Shafique et al., 2022).

The process involved pixel-wise comparison across sequential DAS: 34–41 DAS (7 days) and 34–47 DAS (14 days), identifying temporal transitions between classes. This study will leverage the differencing techniques commonly used in remote sensing (Singh, 1989; Coppin and Bauer, 1996). The mathematical expressions (Equation 10) are used to represent the logical operations executed in R. This expression reflects class transitions between paddy, soil, and weed over two temporal datasets and will be presented using a change matrix table. Therefore, given paddy = 0, soil = 1, and weed = 2, the transition of each class can be calculated using the equation below:


(10) $$Increase/Decrease=\sum_{i,j}\left(\left(P_{i,j}^{\left(t_{1}\right)}=0\;\wedge P_{i,j}^{\left(t_{2}\right)}=2\right)+\left(P_{i,j}^{\left(t_{1}\right)}=1\wedge P_{i,j}^{\left(t_{2}\right)}=2\right)\right)$$

where

$P_{\left(i,j\right)}^{\left(t_{1}\right)}$ = Pixel classification at Time 1

$P_{\left(i,j\right)}^{\left(t_{2}\right)}$ = Pixel classification at Time 2

∧ = Logical AND operator, ensuring both conditions are met simultaneously

$P_{i,j}^{\left(t_{1}\right)}=0\;\wedge \;P_{i,j}^{\left(t_{2}\right)}=2$ = Paddy turned into weed

$P_{i,j}^{\left(t_{1}\right)}=1\;\wedge \;P_{i,j}^{\left(t_{2}\right)}=2$ = Soil turned into weed

∑​i,j (Summation) = Total increase/decrease for paddy, soil, and weed across the image

The method used in this study eliminates reliance on empirical thresholds, often required in traditional vegetation index-based change detection, and instead leverages discrete class transitions to produce actionable insights for site-specific weed management. The complete set of transitions was summarized in a change matrix, providing quantitative measurements of weed expansion, paddy loss, and soil exposure.

Subsequently, the percentage change for each class can be calculated by dividing the number of pixels that have changed by the total number of valid pixels. Mathematically can be calculated using the Equation 11 below:


(11) $$Class\;changes\;\left(\%\right)=\;\frac{Number\;of\;change\;pixels}{Total\;valid\;pixels}\;\times \;100$$

This calculation enables the precise quantification of class-specific changes over time, providing a clear understanding of the temporal dynamics of weed infestation and crop competition. The resulting change percentages serve as key indicators to assess the rate of weed expansion, the corresponding reduction in paddy coverage, and the potential adjustments required for herbicide management interventions.

#### 2.2.5 Herbicide consumption calculation

Calculating the density of weed in the study plots from the weed classification map is important for predicting herbicide consumption and optimizing its usage (Zou et al., 2021). To determine weed density, the number of pixels classified into the designated classes; weed, paddy, and soil will be evaluated. Then, weed density will be calculated as a percentage using Equation 11 below.


(12) $$Weed\;density\;\left(\%\right)=\;\frac{Number\;of\;pixels\;of\;weed}{Total\;number\;of\;all\;pixels}\;\times \;100\%$$

The values of weed density calculated by using Equation 12 above will be used to calculate the area that is being covered by each class in the study area using the Equation 13 below.


(13) $$Coverage\;area=\;\frac{Density\;of\;each\;class\;\times \;Total\;area\;of\;the\;study\;area}{100}$$

Additionally, field investigations and interviews with farmers were conducted to gather information on their herbicide usage for controlling broadleaved weeds in the study plot. Therefore, by using the information provided by the farmers and the percentage of weed density calculated from Equation 12, the estimated amount of herbicide needed can be calculated as shown in Equation 14 below.


(14) $$Estimated\;Hebicide\;\left(ml\right)=\;\frac{\;Weed\;density\;\left(\%\right)\;\times \;total\;herbicide\;used\;by\;farmers\;}{100}$$

Therefore, the expected reduction in herbicide consumption by farmers’ practices based on the zoning map can be calculated using the following Equation 15.


(15) $$Expected\;reduction\;\left(\%\right)=\;\frac{\left(Farmers\;practices-Estimated\;herbicide\right)}{Total\;herbicide\;used\;by\;farmers}\;\times \;100\%\;$$

## 3 Results

**Figure 5:**
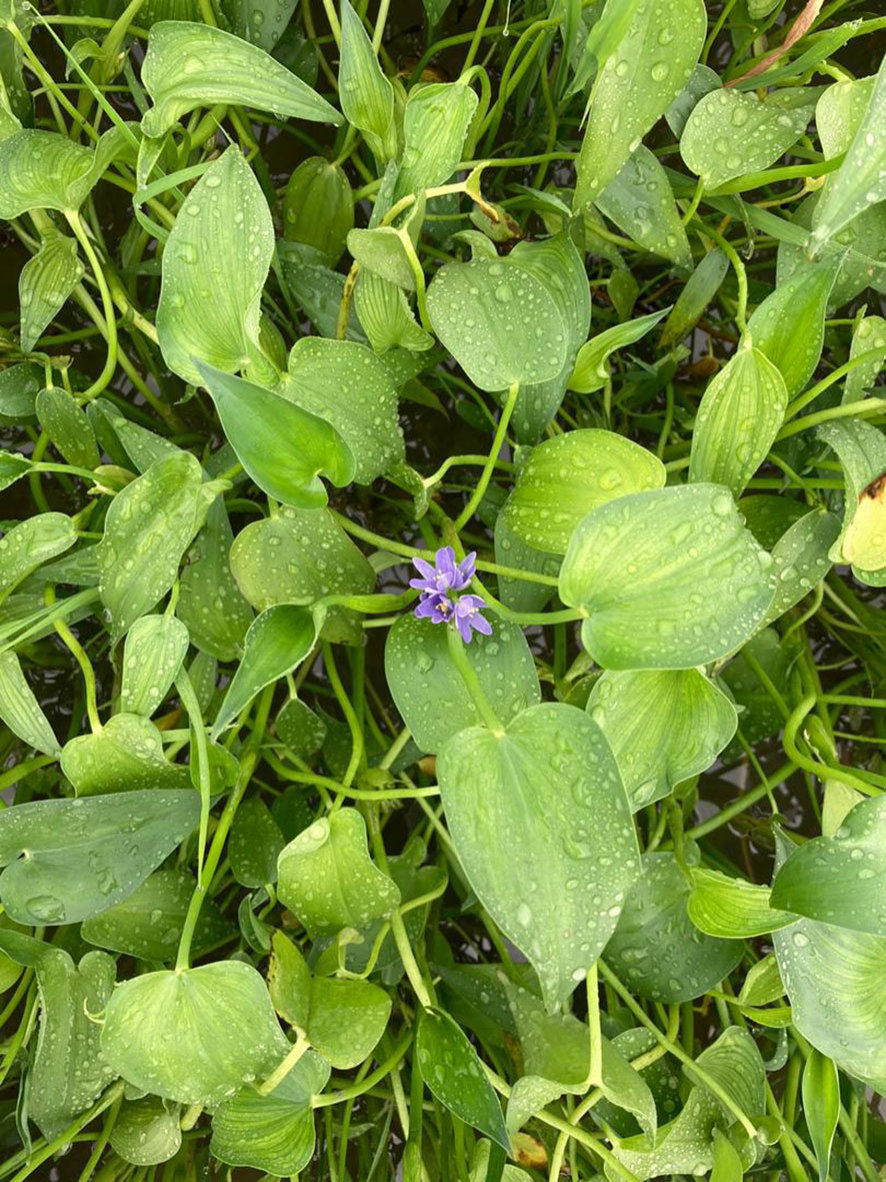
*Monochoria vaginalis* (Burm.f.) C. Presl.

From observation, the study area was dominated by *Monochoria vaginalis* (Burm. f) C. Presl, known as pickerel weed or, in Malay, *keladi agas*. Results showed that in T1 treatment plots, Plot 3, Plot 6, and Plot 8 were highly infested with *M. vaginalis* compared to Plot 1. The investigation reveals that Plot 1 exhibits lower weed distribution compared to the other plots, attributable to its water level of 1 cm. On the other hand, the remaining plots, which are Plot 3, Plot 6, and Plot 8, recorded water levels of 5 cm, 3 cm, and more than 15 cm, respectively. This investigation suggests that there is a possibility that the infestation of *M. vaginalis* in the study plot was highly dependent on the presence of water. Therefore, **Figure 5** shows a picture of *M. vaginalis*, which dominated the study area.

### 3.1 DFNN classification accuracy

Experiments were conducted to assess the model’s performance under two treatments: with treatment (T0) and without treatment (T1). In the T0 plots, the absence of weeds simplified the classification task, while the untreated plots required the model to classify three distinct classes. The model was trained using training samples collected from 34 DAS and 41 DAS datasets. Therefore, **Table 4** summarizes the accuracy, loss, mean square error (MSE), and kappa coefficient at training, testing, and validation of the DFNN model.

With the treatment of herbicide applied (T0), the DFNN model showed high performance across all metrics. Given the model’s need to classify paddy and soil only, the accuracy was incredibly high, with training, validation, and testing accuracies of 0.99, 0.9906, and 0.9803, respectively. The low loss values, which are less than 0.1 (0.0287 for training, 0.0264 for validation, and 0.0224 for testing), show that the model effectively distinguishes between these two classes. Furthermore, the mean squared error (MSE) was also reported as low, ratifying the model’s precision in a less complex environment. The Kappa coefficient reported in **Table 2** was 0.9812, representing almost perfect agreement between actual classifications and model predictions, which aligns with the expected outcome given the reduced classification complexity.

However, the complexity of the classification task in without treatment plots (T1) increased due to the infestation of weeds. Still, **Table 2** shows the training accuracy remained high at 0.9729, with validation and testing accuracies of 0.98 and 0.9793, respectively. Meanwhile, the loss values for training (0.0791), validation (0.067), and testing (0.0597) are also lower than 0.1. In addition, the Kappa coefficient’s value also shows an almost perfect agreement between actual classifications and model predictions, with a value of 0.9789. This implied that the model managed to overcome the challenges caused by the complexity and accurately classified the additional weed class.

In addition to overall accuracy and kappa coefficients, the confusion matrices for both treatment conditions are presented in **Table 5**. These matrices provide the basis for the per-class precision, recall, and F1-score calculations shown in **Table 6**. The model demonstrates strong class separation, with minimal misclassification across all categories.

**Table 6:** Per-class precision, recall and F1-scores for the classification of paddy, soil and weed under with treatment (T0) and without treatment (T1) conditions.

Treatment plot	Class	Precision	Recall	F1-score
T0	Paddy	0.978	0.987	0.983
	Soil	0.986	0.976	0.981
T1	Paddy	1.000	0.998	0.999
	Soil	1.000	1.000	1.000
	Weed	0.998	1.000	0.999

T0 = with herbicide treatment; T1 = without herbicide treatment.

**Precision, recall, and F1−scores are calculated per class from the confusion matrices.

Under T0 plots, both paddy and soil classes achieved high precision with values of 0.978 and 0.986, respectively. Meanwhile for recall, the values of 0.987 (paddy) and 0.976 (soil) were recorded, resulting in balanced F1−scores above 0.98. This indicates that the DFNN model was able to accurately distinguish paddy from soil given that the herbicide treatment reduced weed presence.

Under T1 plots, classification performance was near−perfect across all classes, with precision, recall, and F1−scores ≥ 0.998. Notably, weed’s class achieved a precision of 0.998 and a recall of 1.000, reflecting the model’s ability to detect weed patches with minimal false positives or false negatives in untreated fields where infestation was more pronounced.

### 3.2 Transition dynamic matrices

#### 3.2.1 Seven-day interval

The change matrix shows the transition of land cover classes, paddy, and soil from 34 DAS to 41 DAS (7 days) in pixel counts. Therefore, **Table 7** shows the average transition of the land cover classes for with treatment (T0) plots and **Table 8** for without treatment (T1) plots. Each transition will be color coded for easy visualization, blue for remaining unchanged or no transition, and pink for transition from class paddy to soil and vice versa. Generally, there is a noticeable change from 34 DAS to 41 DAS in T0 and T1 plots.

**Table 7:** Change matrix for T0 plots (seven-day intervals).

34 DAS	41 DAS	
	Paddy	Soil
Paddy	835734	147253
Soil	325579	314488

**Table 8:** Change matrix for T1 (seven-day intervals).

34 DAS	47 DAS		
	Paddy	Soil	Weed
Paddy	840424	62139	106782
Soil	132212	175475	38636
Weed	73212	2024	236840

The transition of paddy’s class in T0 plots shows that the total number of 835,734 pixels has remained as paddy, and 147,253 pixels of paddy have changed to soil. Meanwhile, for soil’s class, the total number of 325,579 pixels has transformed to paddy and 314,488 pixels have remained as soil. Therefore, the soil’s class has the highest transition compared with the paddy classes throughout the T0 plots.

Meanwhile, in T1 plots, the transition of paddy’s class shows that the total number of 840,424 pixels has remained as paddy, 62,139 pixels of paddy have changed to soil, and 106,782 pixels have changed to weed. Meanwhile, for soil class, the total number of 132,212 pixels has changed to paddy, 175,475 pixels have remained as soil, and 38,636 pixels have been transformed into weeds. However, for weed, within seven days, 73,212 pixels have changed to paddy, 2,024 pixels have changed to soil, and 236,840 pixels remain as weed. Therefore, the soil class has the highest transition, followed by the paddy and weed classes, respectively, throughout the T1 plots.

#### 3.2.2 14-day interval

The change matrix shows the transition of land cover classes, paddy, and soil from 34 DAS to 47 DAS (14 days) in pixel counts. Therefore, **Table 9** shows the average of transitions of the land cover classes for with treatment (T0) plots and **Table 10** for without treatment (T1) plots. Each transition will be color coded for easy visualization, blue for remaining unchanged or no transition, and pink for transition from class paddy to soil and vice versa. Generally, there is a noticeable change from 34 DAS to 47 DAS in T0 and T1 plots.

**Table 9:** Change matrix for T0 (14-day intervals).

34 DAS	47 DAS	
	Paddy	Soil
Paddy	929424	71840
Soil	366852	193432

**Table 10:** Change matrix for T1 (14-day intervals).

34 DAS	47 DAS		
	Paddy	Soil	Weed
Paddy	838324	10552	160637
Soil	163361	85253	65366
Weed	56268	725	255076

The transition of paddy’s class in T0 plots shows that the total number of 929,424 pixels has remained as paddy, and 71,840 pixels of paddy have changed to soil. Meanwhile, for soil’s class, the total number of 366,852 pixels has transformed to paddy, and 193,432 pixels have remained as soil. Therefore, within 14 days, the soil’s class has the highest transition compared with the paddy classes throughout the T0 plots.

Meanwhile, in T1 plots, the transition of paddy’s class shows that the total number of 838,324 pixels has remained as paddy, 10,552 pixels of paddy have changed to soil, and 160,637 pixels have changed to weed. Meanwhile, for soil’s class, the total number of 163,361 pixels have changed to paddy, 85,253 pixels have remained as soil and 65,366 pixels have transformed to weed. However, for weed, within 14 days, 56,268 pixels have changed to paddy, only 725 pixels have changed to soil, and 255,076 pixels remain as weed. Therefore, soil class has the highest transition, followed by the paddy and weed classes, respectively throughout T1 plots.

### 3.3 Dynamic changes in vegetation and soil coverage

Seven legends have been created in order to represent the changes. They are No change in paddy, Paddy to soil, Paddy to weed, Soil to paddy, No change in soil, Soil to weed, Weed to paddy, Weed to soil, and No change in weed.

#### 3.3.1 With treatment (T0) plots

**Table 11** shows the transition of paddy and soil classes from 34 DAS to 41 DAS to 47 DAS visually and statistically. In general, there are significant changes in paddy and soil’s classes in the with treatment (T0) plots over time. The percentage of increase and decrease for paddy and soil classes is also presented in **Table 11**. In general, there are significant changes in paddy growth for all plots in T0 treatment.

**Table 11:** The transition dynamics from 34 DAS to 41 DAS to 47 DAS for with treatment (T0) plots.

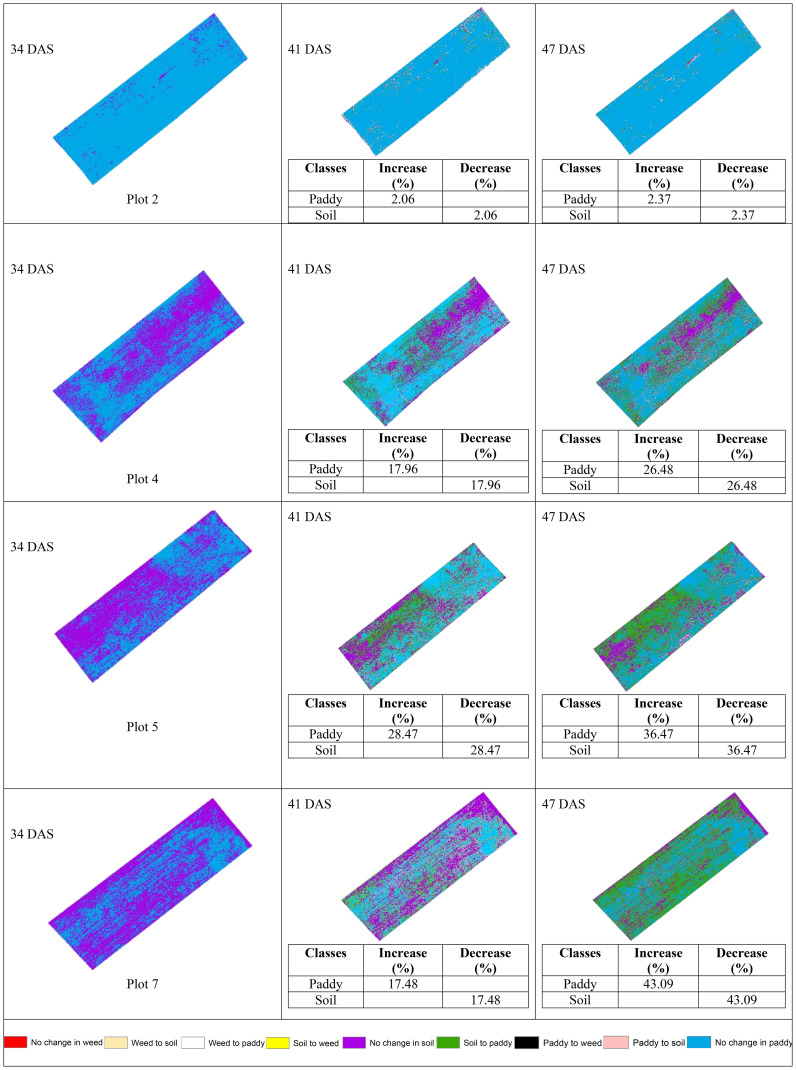

**Table 11** illustrates the spatial changes in paddy and soil coverage over time. Within seven days, Plot 5 has the highest transition for Paddy’s classes (28.47%), and after 14 days, Plot 7 has the highest transition in Paddy’s classes with a 43.09% increase. However, the least changes occur in Plot 2. Within seven days, Paddy’s classes increased by 2.06%, and after 14 days, the increase rate only reached 2.37%. However, this substantial increase in paddy coverage shows that the treatment applied did promote paddy’s growth.

**Figure 6:**
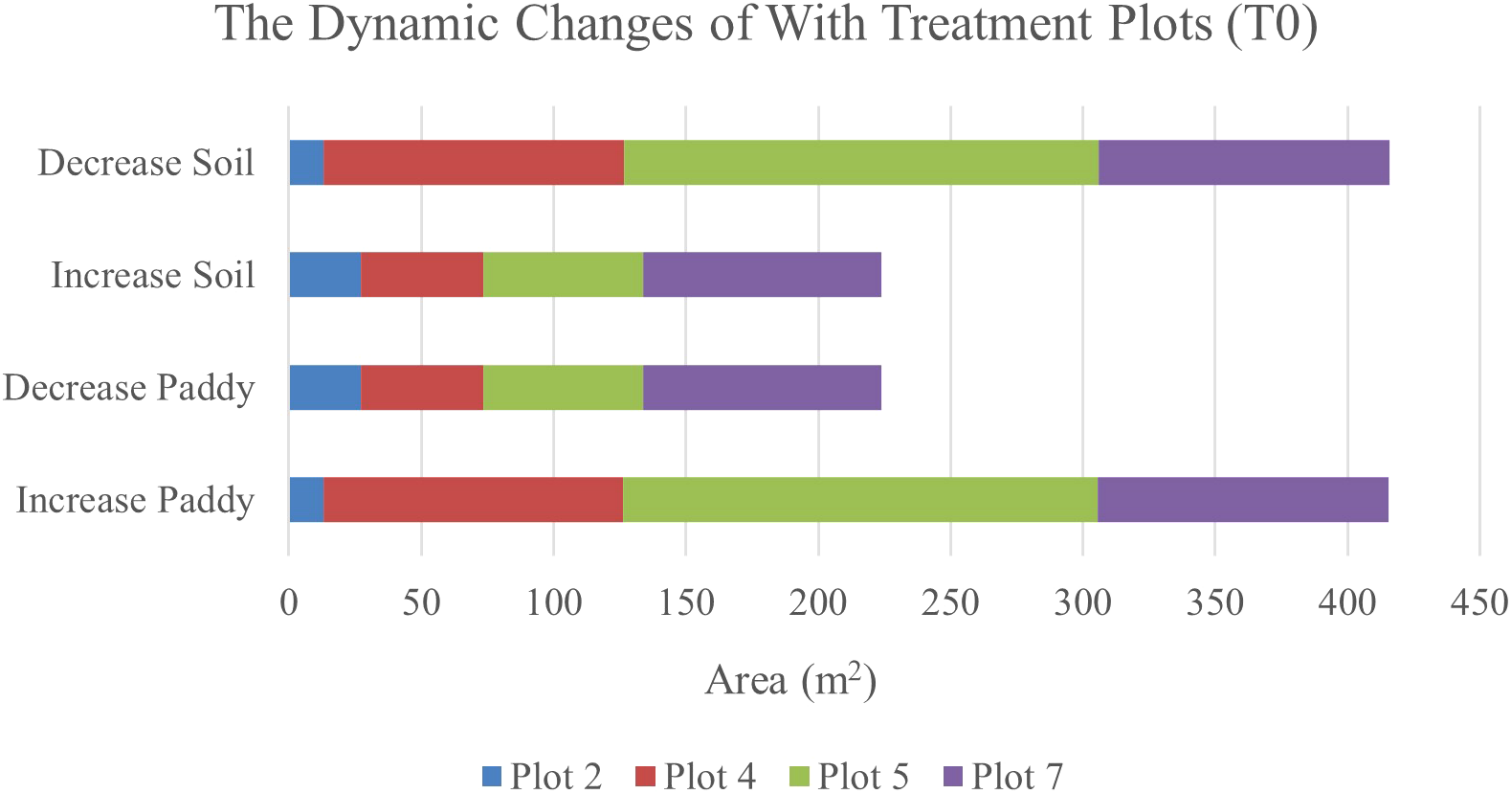
The dynamics changes in area coverage (m^2^) for with treatment (T0) Plot from Week 1 to Week 2 (seven-day intervals).

**Figure 7:**
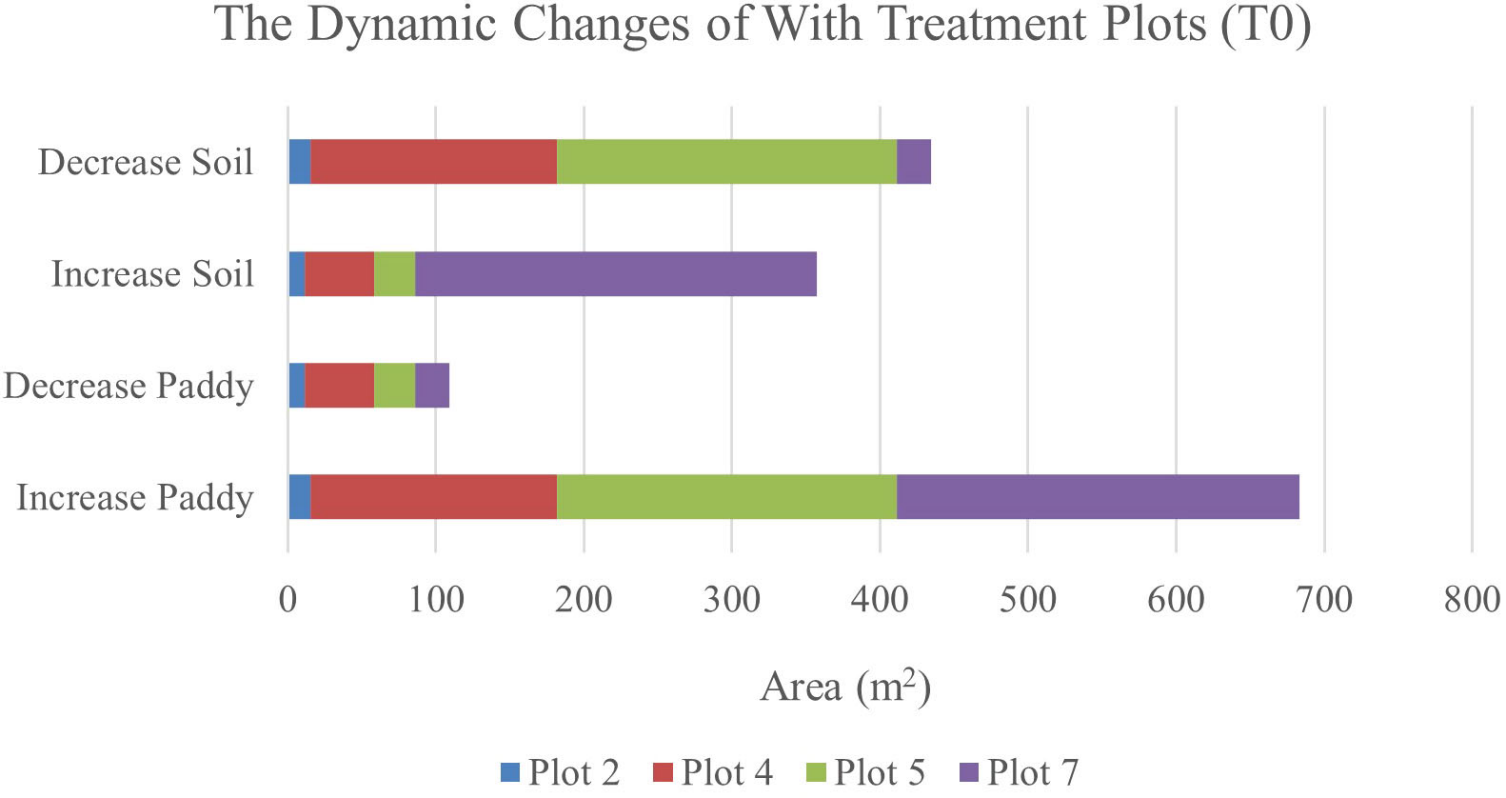
The dynamics changes in area coverage (m^2^) for with treatment (T0) Plot from Week 1 to Week 3 (14-day intervals).

Therefore, **Figure 6** and **Figure 7** will complement these findings by quantifying the land changes into stacked bar charts. The graphs illustrate that the treatment (T0) has led to more stable and predictable changes in vegetation and soil coverage due to less competition with weeds.

#### 3.3.2 Without treatment (T1) plots

**Table 12** shows the transition of paddy, soil, and weed classes from 34 DAS to 41 DAS to 47 DAS, visually and statistically. In general, there are significant changes in paddy, soil, and weed classes in the without treatment (T1) plots over time. The percentage of increase and decrease for paddy, soil, and weed classes is also presented in **Table 12**.

**Table 12:** The transition dynamics from 34 DAS to 41 DAS to 47 DAS for without treatment (T1) plots.

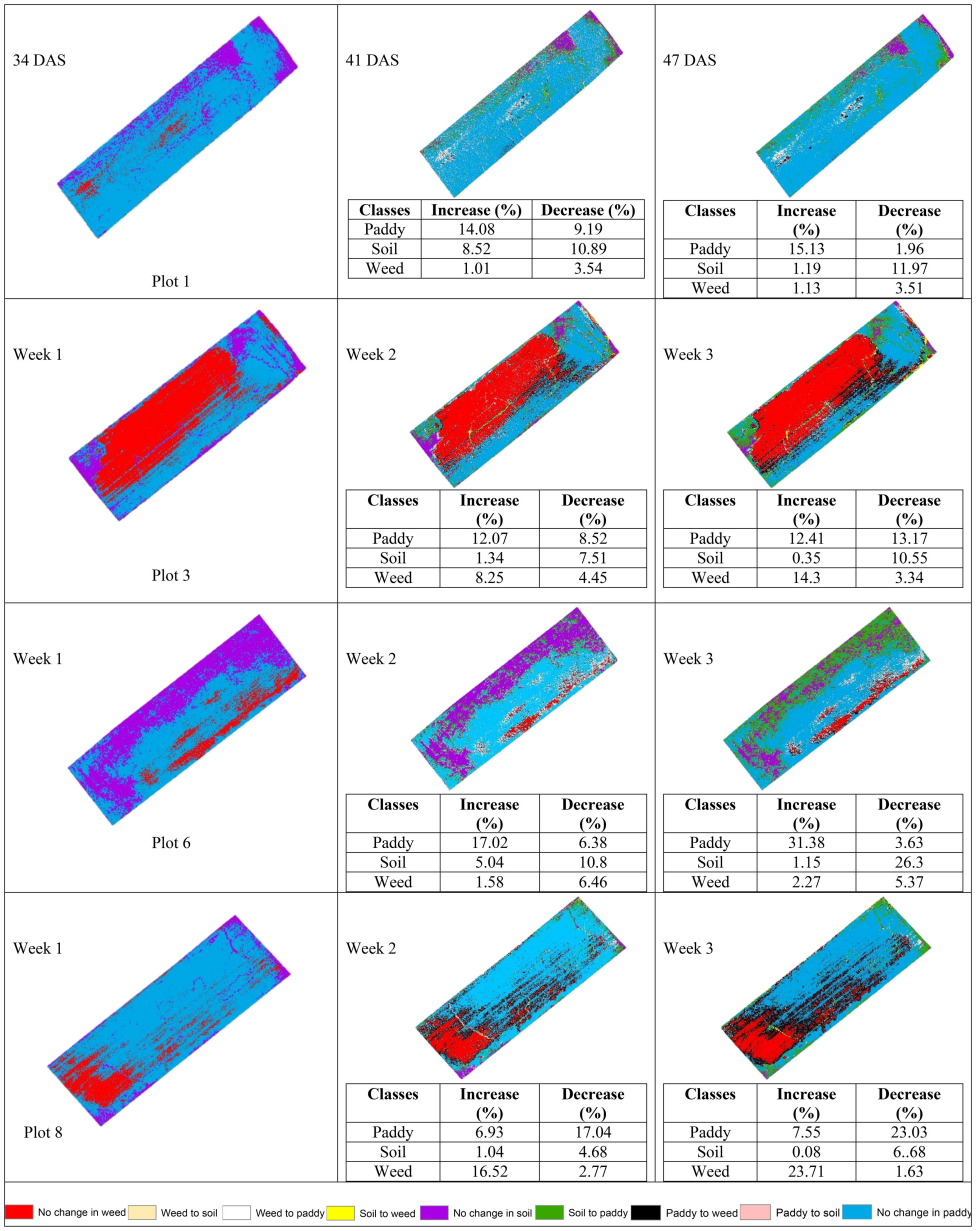

**Table 12** illustrates the spatial changes in paddy, soil, and weed coverage over time. Within seven days, Plot 8 demonstrated the highest changes for paddy and weed classes, where paddy had decreased to 17.04% meanwhile, weed increased by 16.52%. However, in Plot 1, and Plot 6, the increase in weed class is significantly lower, 1.01% and 1.58%, respectively whereas paddy increases up to 14.08% and 17.02%, respectively.

**Figure 8:**
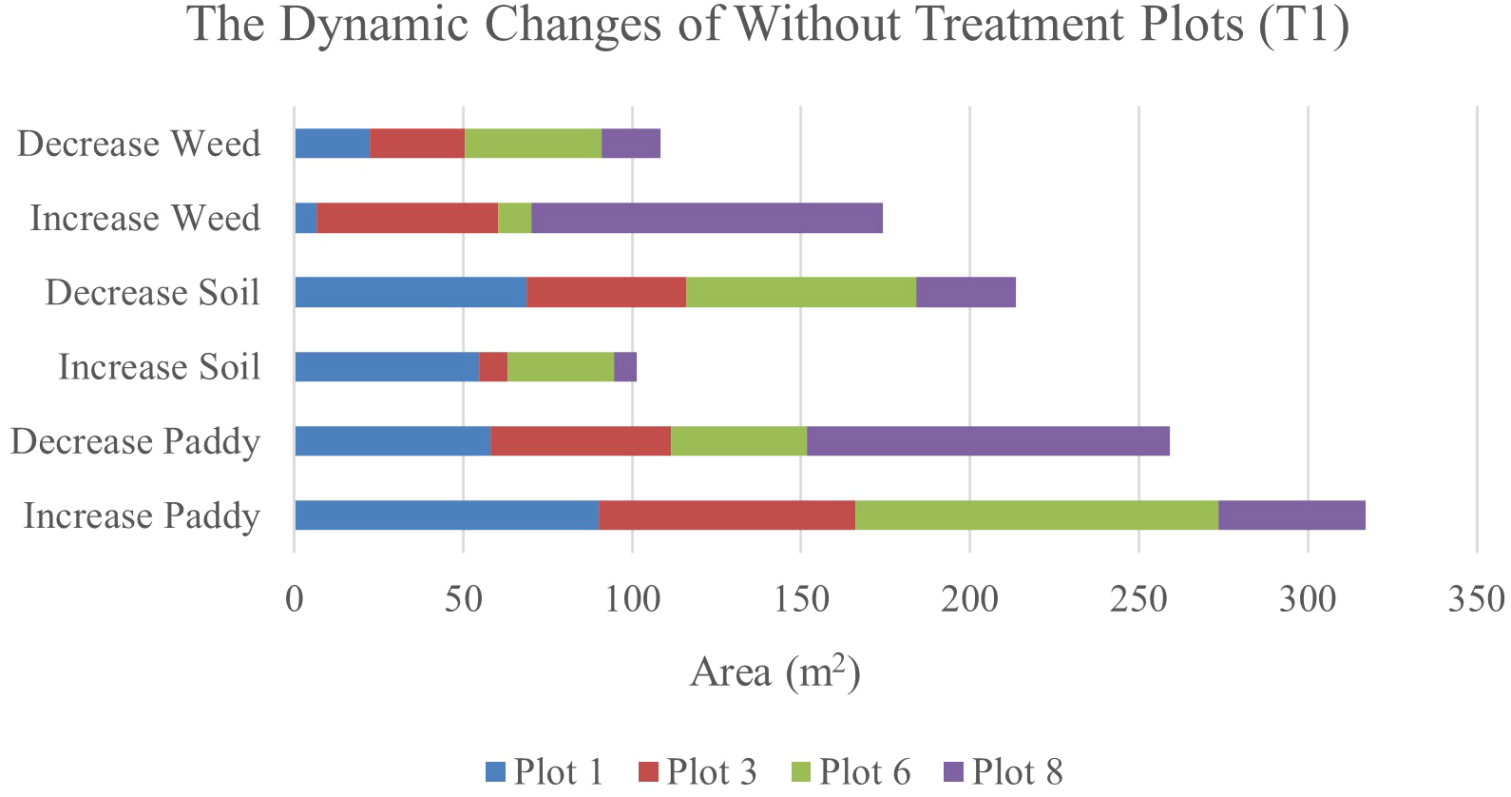
The dynamics changes in area coverage for Treatment (T1) Plot from 34 DAS to 41 DAS (seven days intervals).

After 14 days, weed changes in Plot 8 had increased to 23. 71% and paddy had decreased to 23.03%. Meanwhile, in Plot 3, weed had increased from 8.25% to 14.3% and reduced paddy by about 13.17%. However, similar to 41 DAS, at 47 DAS, Plot 1 and Plot 6 recorded the least increments in weed with values of 1.13% and 2.27%, respectively whereas paddy increased to 15.13% and 31.38%, respectively. This pattern implies a possible change from exposed soil to paddy growth over the study period and these two plots are not providing a sustainable environment for weeds to grow. Therefore, **Figures 7**, **8** will complement these findings by quantifying the land changes into stacked bar charts.

In the seven-day interval chart (**Figure 8**), the most noticeable changes are seen in the increase of weed coverage across most plots. Plot 8 shows a significant increase in weed coverage, about 149.37m^2^, while paddy and soil areas had decreased to 145.09m^2^ and 42.08m^2^, respectively. This change indicates a rapid growth rate of weeds in the Without Treatment (T1) plots, which influences the overall paddy growth by raising competition for nutrients and space. In contrast, Plots 1 and 6 displayed a small area of weed coverage and most of the area in these two plots is transited to soil and paddy. This trend indicates possible variations in variability due to water content.

**Figure 9:**
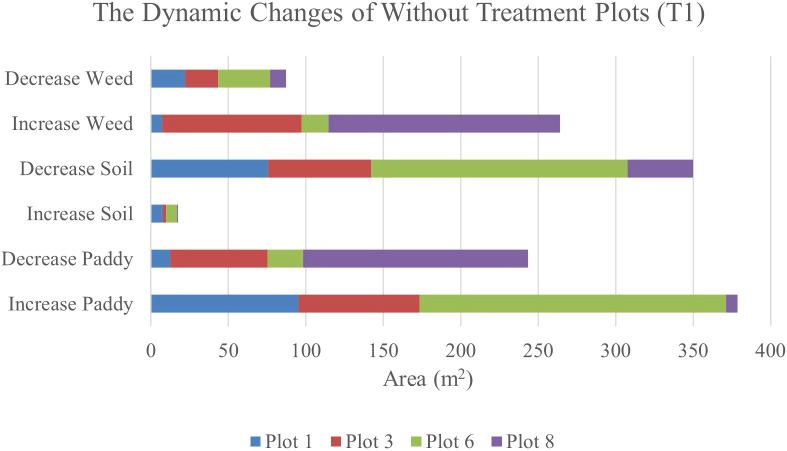
The dynamics changes in area coverage (m^2^) for without treatment (T1) Plot from 34 DAS to 47 DAS (14 days intervals).

When the observation was extended to 14 days, the dynamic changes in weed infestation became more noticeable. In this stacked bar chart (as shown in **Figure 9**), Plots 3 and 8 experienced significant increases in weed coverage, with values of 90.09m^2^ and 149.37m^2^, respectively compared to the seven-day interval with values of 53.68m^2^ and 104.8m^2^, respectively. These changes indicate that the severity of weed infestation increases with time when left untreated. Meanwhile, for paddy, its coverage exhibited both increases and decreases. These trends reflect the varying growth rates and competitive pressures exerted by weeds. However, a decrease in soil coverage is expected. Active growth by paddy and weed takes all open space in the study plots.

### 3.4 Change detection maps

**Figure 10:**
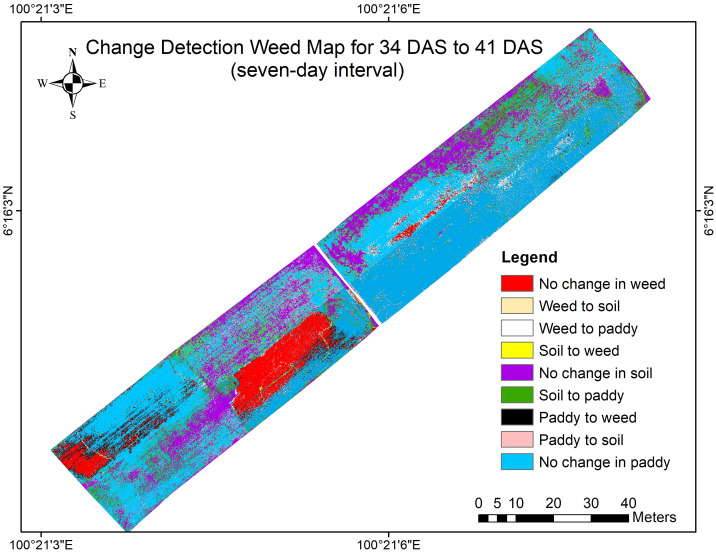
The change detection map for 34 DAS to 41 DAS (seven-day intervals).

Chang detection maps were divided into nine legends which are No change in paddy, Paddy to soil, Paddy to weed, Soil to paddy, No change in soil, Soil to weed, Weed to paddy, Weed to soil, and No change in weed. Therefore, **Figure 10** shows the change detection map for 34 DAS to 41 DAS (seven-day intervals).

**Figure 11:**
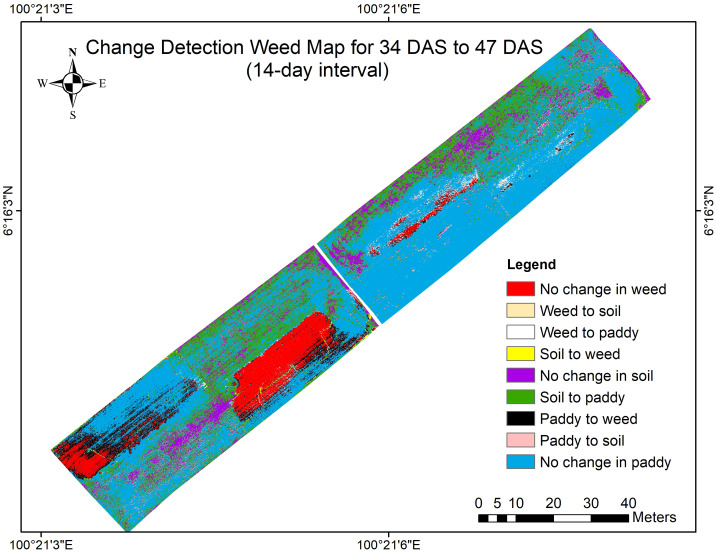
The change detection map for Week 1 to Week 3 (14-day intervals).

This map highlights the early-stage dynamics observed within the seven-day intervals, from 34 DAS to 41 DAS. This change shows the initial response of the weed infestation in the untreated plot in various conditions and how this species suppresses the paddy growth and takes the soil’s open space. The spatial distribution of changes in both treated and untreated plots is visible, supporting the detailed analysis presented earlier. To further investigate the severity of weed infestation, the observation was prolonged at 14-day intervals. Thus, **Figure 11** shows the change detection map for 34 DAS to 41 DAS (14-day intervals).

This map offers a wider perspective of changes over two weeks, demonstrating the persistent progression or stabilization of vegetation classes especially the transition of paddy to weed in Plot 3 and Plot 8. The patterns observed here confirm the trends discussed in the previous subsections, particularly the severity of weed infestation increases over time when left untreated. Therefore, **Table 13** shows the changes in the area of coverage of each class, paddy, soil, and weed that represent the whole study area at 31 DAS, 41 DAS, and 47 DAS. Since this distribution represents the whole study area, the area of coverage was measured in hectares (ha).

**Table 13:** The changes in area of coverage (ha) of each class, paddy, soil, and weed on different Days After Sowing (DAS).

Class/DAS	34 DAS	41 DAS	47 DAS
Paddy	0.31	0.33	0.38
Weed	0.05	0.054	0.07
Soil	0.15	0.12	0.06

**Figure 12:**
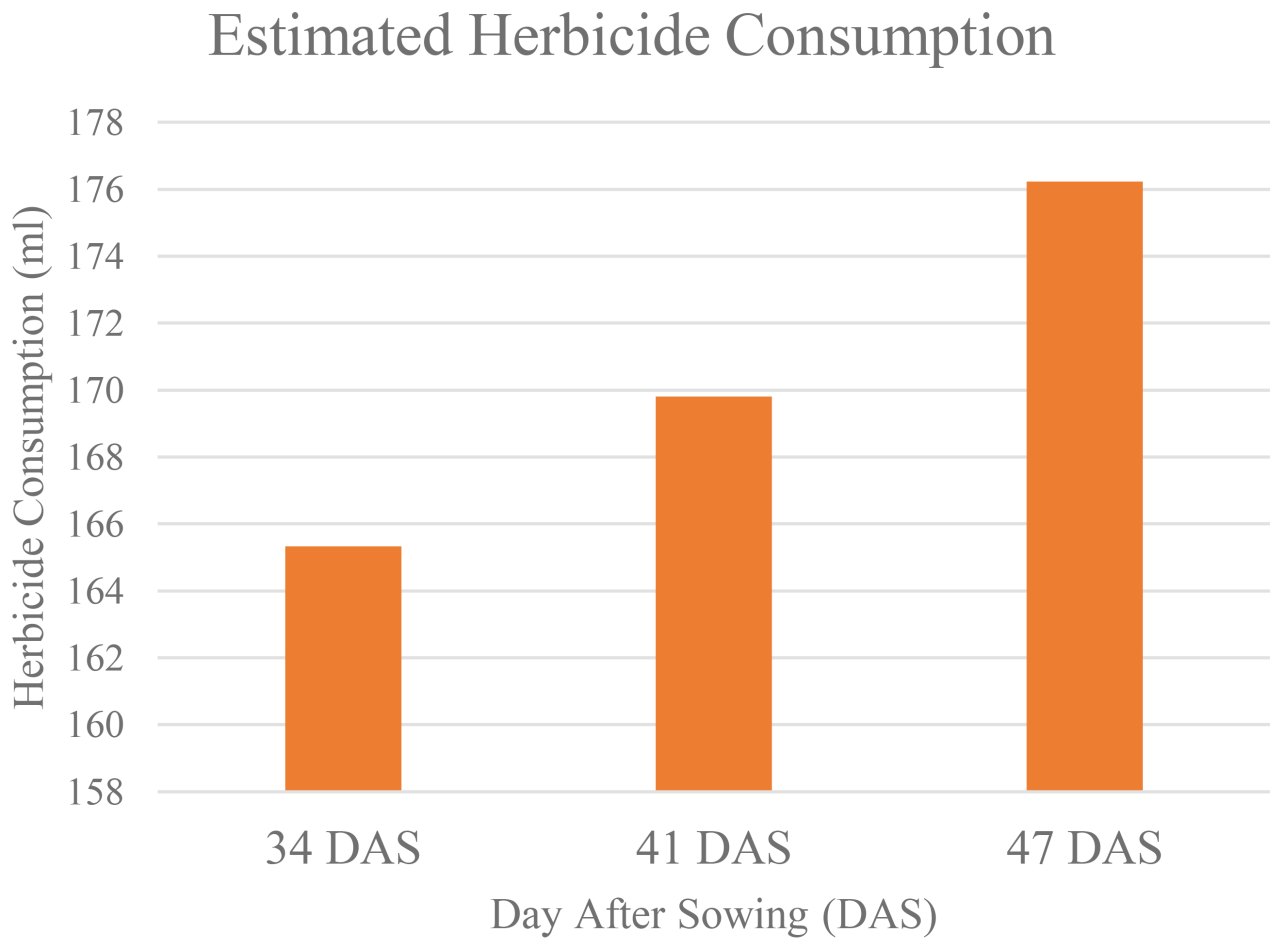
Estimated herbicide consumption over time.

**Table 13** shows that as increasing in number of Days After Sowing (DAS), the area of weeds that infestated the study area also increased. This has caused the estimated of herbicides needed to control weeds to increase (as shown in **Figure 12**).

**Figure 13:**
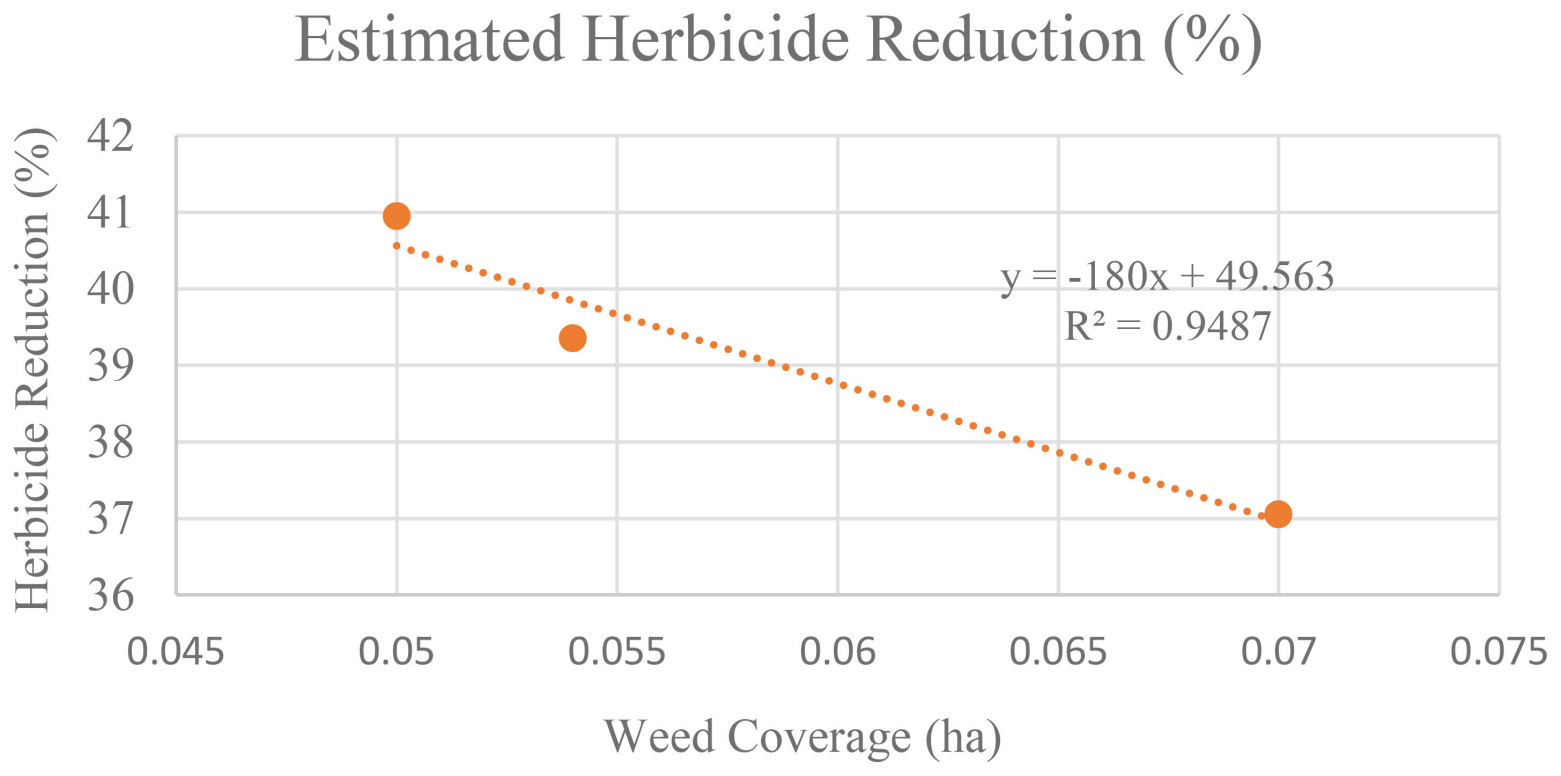
Relationship between the increases in weed coverage with the estimated herbicide reduction.

At 34 DAS, the estimated herbicide needed to control weed is 165.33ml. However, when weed was left untreated, within seven days, the amount increased to 169. 803ml, and after 14 days, it increases to 176.23ml. This makes the estimated herbicide reduction decrease. At 34 DAS, farmers were expected to reduce herbicide usage by up to 40.95%. But, as the area of weed coverage increased, at 41 DAS and 47 DAS, the reduction in herbicide usage decreased to 39.36% and 37.06% respectively. Thus, **Figure 13** establishes the relationship between weed infestation over time with the estimated herbicide reductions.

**Figure 13** illustrates the inverse relationship between weed coverage increase and estimated herbicide reduction. The regression graph forms a near-perfect straight line, with an R² value of 0.9487, indicating a strong relationship between these two parameters. This strongly suggests that as weed coverage increases over time when left untreated, the potential reduction in herbicide usage decreases significantly.

## 4 Discussion

This study successfully utilized multispectral UAV imagery with a spatial resolution of 0.913 cm to monitor weekly changes in weed infestation within a paddy field. A deep feedforward neural network (DFNN) was employed to classify paddy, weed, and soil in both with (T0) and without treatment (T1) plots. The differencing approach revealed significant changes in weed and paddy cover over time. In just seven days, weed cover increased up to 16.52%, while paddy cover decreased by 17.04%. After 14 days, weed cover increased to 23.71%, suppressing paddy to 23.03%. These changes indicate a growing weed infestation negatively impacts crop health and yield when left untreated.

The differencing technique for change detection successfully identified transitions between paddy, weed, and soil classes at seven and 14-day intervals. This approach works by calculating the difference in pixel values between two dates, which highlights areas of significant change, as demonstrated in Section 3.2. This outcome is consistent with Ke et al. (2018), who also found that the differencing method successfully distinguished the changed and unchanged pixels in two different images. As Maimaitijiang et al. (2020) suggested, high-resolution UAV imagery provides more precise monitoring than satellite imagery, which is critical for detecting minor changes in weed growth and crop health. In this study, the UAV’s spatial resolution of 0.913 cm at a flying height of 20m allowed for the identification of even small-scale changes in the field.

As shown in **Table 11**, a high growth rate was expected for paddy in the treatment (T0) plots, where there was no competition for space and nutrients with weeds. Research by Pereira et al. (2022) shows that the application of herbicides such as 2,4-D, Lactofen, and Imazetapyr is necessary to improve and increase crop productivity. Therefore, monitoring weed infestation trends is essential to enable targeted weed control measures. However, the observed transitions, such as soil being reclassified as paddy, are most likely attributable to canopy closure during rice tillering, when emerging leaves cover previously exposed soil. Nevertheless, a degree of classification uncertainty cannot be ruled out, and such transitions should be interpreted with caution.

**Table 12** shows that, within the first seven days (34 DAS to 41 DAS), the infestation rate varied significantly across the plots, with the highest rate in Plot 8 (17.04%), moderate infestation in Plot 3 (8.52%), and minimal increases in Plot 1 (1.01%) and Plot 6 (1.56%). By the 14th day (47 DAS), the infestation rates nearly doubled in Plot 3 and Plot 8, reaching 14.3% and 23.71%, respectively. Meanwhile, Plots 1 and 6 saw only slight increases. These trends align with the water levels observed as recorded in the result section. This suggests that water management directly impacts weed growth dynamics. Proper water management plays a critical role in controlling weed growth, with higher water levels providing favorable conditions for broadleaved weeds, especially for *M. vaginalis* species (Setiawan and Sintadevi, 2021).

In addition, in untreated plots, change detection analysis shows that, when weed infestation increases over time, the estimated herbicide demand also increased (**Figure 12**). Hence, farmers were expected to reduce herbicide usage by 40.95%, but as weed coverage spread out, this reduction decreased to 39.36% at 41 DAS and further to 37.06% at 47 DAS. Therefore, **Figure 13** demonstrated a strong inverse relationship between weed infestation over time with the estimated herbicide reductions with R^2^ values of 0.9487. The unchecked growth of weeds not only increased the need for herbicides but also decreased the potential values of early reduction strategies (Peerzada et al., 2019).

To mitigate the negative impacts of weed infestation, it is recommended to implement weed control measures at or before 42 DAS. At this stage, weeds can effectively compete with paddy for essential resources like nutrients and sunlight, leading to yield losses ranging from 10% to 83% (Kaur et al., 2022). However, according to Lakra et al. (2022), crop growth and yield productivity can be improved by applying the herbicide at 35 DAS which aligns with our analysis, 34 DAS is the optimal time for weed intervention before the rapid development of weed growth observed between 34 and 47 DAS.

This finding is also consistent with what’s been practiced by farmers in Malaysia. As recorded in the Rice Check Padi (DoA, 2022), for rice varieties that mature at 100 DAS, herbicide application should be completed before 40 DAS. Meanwhile, for rice varieties that mature at 125 DAS, herbicide should be applied before 60 DAS. In this study, the PadiU Putra variety, which matures at 120 DAS (Berahim et al., 2021), was used. The agreement between our analysis and these guidelines indicates that the optimal intervention period identified in this study is adaptable to be applied across both early- and late-maturing rice varieties. This demonstrates the broader application of our findings, suggesting that timely weed control interventions before or at 34 DAS can benefit rice cultivation regardless of the variety.

Recent studies have demonstrated the effectiveness of UAV-based deep learning models for weed detection across various cropping systems. For example, Castellano et al. (2023) employed lightweight Vision Transformers on multispectral imagery, achieving high segmentation accuracy (OA: 94.6%, Kappa: 0.91, F1-score: 92.8%) using the WeedMap dataset. Similarly, Liu et al. (2024) applied deep spectral analysis to multispectral UAV data in wheat fields, reporting precision scores above 91% and quantifying significant yield losses due to weed infestation. Seiche et al. (2024) compared high-end and low-cost multispectral sensors using a U-Net architecture, with F1-scores ranging from 76% to 82%, underscoring the influence of sensor quality on detection performance.

In contrast, the present study introduces a Deep Feedforward Neural Network (DFNN) specifically tailored for structured, non-spatial, pixel-wise multispectral data in Malaysian rice fields. The DFNN achieved strong classification performance, with an overall testing accuracy of 99.06%, Kappa coefficients of 0.9812 (T0) and 0.9789 (T1), and class-specific F1-scores reaching 0.999 for weed detection under untreated conditions. These results underscore the model’s robustness and precision in site-specific weed monitoring. Compared to transformer-based and convolutional architectures, DFNN offers computational simplicity and scalability, making it a promising candidate for near real-time agricultural applications.Beyond the local context, the findings of this study have broader implications for global weed management practices and sustainable agriculture. By showing how high-resolution UAV imagery integrated with DL can effectively monitor weed infestation trends, this research contributes to the advancement of scalable, data-driven approaches for site-specific weed control. More importantly, early detection and timely intervention can aid in optimizing herbicide usage, decreasing environmental contamination, and upholding long-term soil health providing to climate-resilient agriculture (Sarma et al., 2024). These outcomes directly support global food security efforts and align with key Sustainable Development Goals (SDGs), particularly SDG 2: Zero Hunger, SDG 12: Responsible Consumption and Production, and SDG 13: Climate Action (Schröder et al., 2019; Hughes, 2020; Filho et al., 2023). With further clarification and integration into national-level precision agriculture (PA) initiatives, this approach has the potential to aid both sustainability and productivity in rice farming organizations globally.

### 4.1 Limitations and future recommendations

It should be noted that the study area was dominated by *M. vaginalis*. The dominance of this weed in flooded plots aligns with its well-documented aquatic ecology (Hazrati et al., 2023). Its broad leaf surface area and enhanced photosynthetic efficiency allow it to establish early and aggressively compete with rice plants during the critical vegetative stage (Gao et al., 2023). As the primary objective of this study was to monitor broadleaved weed infestation, the DFNN was designed and validated within this scope. While the model demonstrated strong classification performance for *M. vaginalis*, further validation across fields infested with other weed functional groups, such as grasses and sedges, is needed to evaluate its generalizability and transferability. In addition, future studies should investigate whether classification transitions such as “weed to paddy” or “soil to paddy” reflect true ecological processes such as, crop canopy expansion, weed suppression or potential misclassification due to spectral ambiguity. This will require integrating field-level observations and temporal consistency checks to improve interpretability and model reliability.

In addition, we also observed a potential correlation between water level variation and weed distribution. This may explain why *M. vaginalis* thrives under waterlogged conditions, such as in paddy fields, making it especially difficult to manage. Since water depth was not explicitly controlled in the experimental design, these observations remain correlative rather than causal. Therefore, future studies should incorporate water management as an experimental variable. This could provide stronger evidence of its role in weed infestation dynamics. Moreover, water depth may influence spectral reflectance and contribute to classification transitions that are difficult to interpret without supporting ecological data (Singh et al., 2023). Incorporating water level monitoring could help distinguish between genuine vegetation changes and classification artifacts, especially in flooded environments.

In this study, herbicide saving potential was estimated under the simplifying assumption of a linear relationship between weed coverage and herbicide requirement. However, in practice, factors such as weed density, species composition and growth stage may influence herbicide efficacy. Therefore, this estimation should be regarded as a first-order approximation. Future research should refine this relationship through dose–response trials and agronomic validation in order to improve its practical applicability. Additionally, understanding how classification transitions such as weed to soil or weed to paddy can be related to actual weed suppression. This could enhance the ecological relevance of herbicide planning. Linking spectral transitions to field-level weed dynamics will be essential for developing more precise intervention strategies. Finally, future studies should aim to increase the temporal resolution of UAV data acquisition to better capture the dynamics of weed infestation and crop development stages. While this study was limited to three key time points due to COVID-19 restrictions, denser temporal sampling would enable a finer analysis of vegetation transitions and enhance the robustness of temporal change detection models. This study used a DFNN trained from scratch. Therefore, future work could explore the use of transfer learning or pre-trained CNN backbones fine-tuned on multispectral or hyperspectral data to potentially boost classification accuracy and training efficiency, especially in limited-label scenarios.

## 5 Conclusion

This study successfully demonstrated the effectiveness of multispectral UAV imagery combined with deep feedforward neural networks to track the growth rate of weed infestation in paddy fields using change detection analysis. Within 7 days (34 DAS to 41 DAS), the growth rate of weed is 16.52%. However, over 14 days, the weed growth rate increased significantly, reaching 23.71%, while paddy cover decreased by 23.03% in untreated plots. On 34 DAS, farmers expected a 40.95% herbicide reduction. However, within 14 days, the reduction decreases to 37.06%, amounting to a total reduction of 3.89% over 14 days. A strong inverse relationship between weed infestation over time and estimated herbicide reductions was established, with a high R² value, 0.9487. This relationship highlighted that early detection and control are crucial, as unchecked weed growth led to a higher demand for herbicides and reduced the potential for early herbicide reduction strategies. Timely intervention, especially before 34 DAS, is essential to mitigate weed competition and preserve crop yields.

## Data Availability

The original contributions presented in the study are included in the article/supplementary material. Further inquiries can be directed to the corresponding author.
